# Synthesis and X‑ray Structures of Bis-Functional Resorcinarene Crown Ethers

**DOI:** 10.1021/acs.cgd.5c00295

**Published:** 2025-06-25

**Authors:** Frank Boateng Osei, Sanaz Nadimi, Jas S. Ward, Sarah Nasri, Abd al-Aziz A. Abu-Saleh, Elham Pourian, John F. Trant, Kari Rissanen, Ngong Kodiah Beyeh

**Affiliations:** † Department of Chemistry, Oakland University, 146 Library Drive, Rochester, Michigan 48309-4479, United States; ‡ Department of Chemistry and Biochemistry, University of Windsor, 401 Sunset Avenue, Windsor, Ontario N9B 3P4 Canada; § Department of Chemistry, University of Jyvaskyla, Survontie 9 B, Jyväskylä FI-40014, Finland; ∥ Binary Star Research Services, LaSalle, Ontario N9J 3 × 8, Canada; ⊥ WE-Spark Research Institute, 401 Sunset Ave, Windsor, Ontario N9B 3P4, Canada; # Department of Biomedical Sciences, University of Windsor, 401 Sunset Ave, Windsor, Ontario N9B 3P4, Canada

## Abstract

Two resorcin[4]­arene-crowns were synthesized with crown ether substituents as the lower rim functionalities, with either the Crown-6 (**TRC6**) or Crown-7 (**TRC7**) moieties being incorporated. X-ray crystallographic data show that both molecules prefer the boat conformation in the solid state. The crown ethers were observed in an askew *anti*-arrangement in **TRC6** and a *syn*-arrangement in **TRC7**. **TRC6** crystallized with four DMF and four H_2_O molecules. Two of the DMF solvent molecules are hydrogen bonded to the resorcinolic OHs, and the other two are entrapped as a dimer between two ether moieties from adjacent **TRC6** molecules. In **TRC7**, four intramolecular hydrogen bonds from the same resorcinol OH group to the crown ether oxygens are observed, with O···O contact distances of 2.742(2) Å and 2.872(2) Å and O–H···O angles of 169.8° and 175.4°. Host–guest binding of potassium and rubidium cations as their chloride and tetrafluoroborate salts in highly competitive DMSO shows 1:1 and 1:2 host–guest complexes for **TRC6** and **TRC7**, respectively. More intense NMR signal changes were observed in less competitive acetonitrile solvent with the tetrafluoroborate salts.

## Graphical abstract

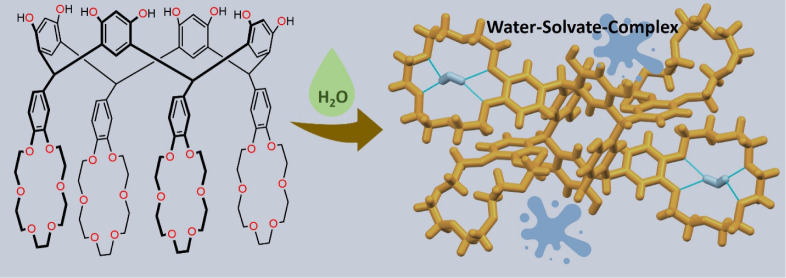

## Introduction

Multicomponent self-assembly is a highly efficient method for achieving structured higher order supramolecular architectures by incorporating many small building blocks through noncovalent interactions,^1,2^ Recently, molecular networks^1,3–5^ composed of many different molecules have been introduced to increase the complexity^6–12^ of chemical systems and to achieve more complex functions. Resorcinarenes are attractive building blocks in supramolecular chemistry possessing a multipurpose scaffold with the potential for further functionalization at both rims.^13,14^ Resorcinarenes can in principle exist in four different configurations at the methine bridges, *i.e*., the *cis*-*cis*-*cis* (*rccc*), *cis*-*cis*-*trans* (*rcct*), *cis*-*trans*-*trans* (*rctt*), and the *cis*-*trans*-*cis* (*rctc*).^15^ The conformations of the macrocycle can adopt several arrangements such as crown (*C*_4*v*_), boat (*C*_2*v*_), chair (*C*_2*h*_), diamond (*C*_s_), and saddle (*D*_2*d*_).^16–23^ There are reports of the rarely observed *rcct*-diamond isomer,^24^ the thermally stable and asymmetric *rcct*-boat stereoisomer^25^ and the C_4t_-pyrogallarene (4t = *tert*-butyl) in the *rcct*-crown conformation.^26^ With no or small C-2 groups in the upper rim and moderately sized alkyl groups (greater than ethyl) in the lower rim, the *C*_4*v*_ conformer tends to dominate as it allows for the formation of a strong hydrogen bond network between the hydroxyl groups of the upper rim; however, tetraalkylation (or more up to full octa/peralkylation) and/or acylation of the resorcinarene hydroxyl groups breaks the cyclic array of intramolecular hydrogen bonds undergoing a conformational change from the common crown (*C*_4*v*_) to boat (*C*_2*v*_).^27^

Resorcinarenes with attached crown ethers can combine two different binding motifs into one molecular entity. Resorcinarene crown ethers utilizing the synthetic architecture of the resorcinarene upper rim such as tetramethoxy *bis*-crown ethers with C4 and C5 ^28^ and tetramethoxy resorcinarene *bis*-crowns *m-* and -*p*-tribenzo-*bis*-crown 6 with aromatic functionality in the crown ether bridge have been reported.^29^

This contribution presents the synthesis of two resorcinarene tetracrowns with four benzo-18-crown-6 (**TRC6**) or four benzo-21-crown-7 (**TRC7**) ethers at the lower rims (Figure 1). The conformation of the resorcinarene skeleton of this divalent host has a pivotal influence on the binding of alkali metals. The conformation of the two resorcinarene tetracrowns (**TRC6** and **TRC7**) are investigated in the solid state through single crystal X-ray crystallography. The binding assemblies with potassium and rubidium (Figure 1) are investigated in solution by NMR spectroscopy and isothermal titration calorimetry (ITC).

**Figure 1:**
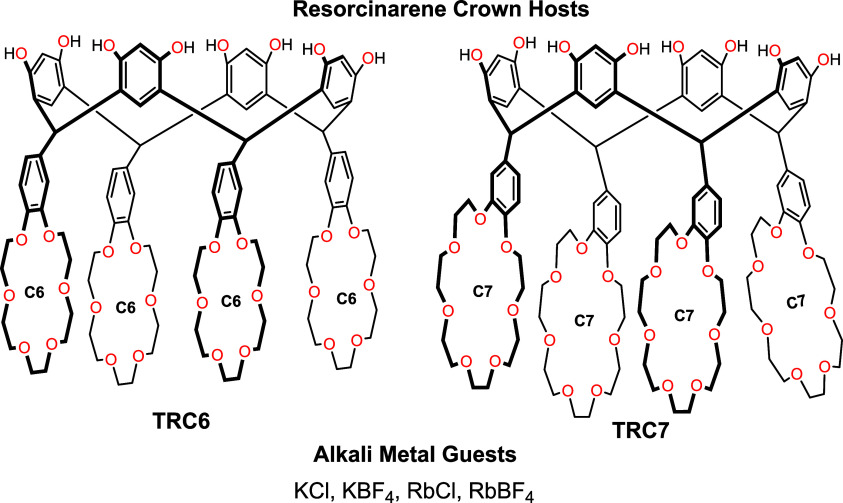
Resorcinarene crown ether hosts (**TRC6** and **TRC7**) and alkali metal (K^+^ and Rb^+^) salt guests.

## Results and Discussion

To synthesize the resorcinarene crown hosts, we first made benzaldehyde crown ethers **C6** and **C7** from 3,4-dihydroxybenzaldehyde **1** by using the protocol of D’Souza and colleagues (Figure 2).^30,31^ Resorcinarenes can be efficiently synthesized in reasonable to high yields using straightforward, single-step methods that do not require templates or high dilution techniques.^13,32^ Benzo-crown ether **C6** or **C7** was added dropwise to a solution of resorcinol in ethanol, water, and HCl at 0–5 °C under nitrogen. After addition of the aldehyde, the mixture was left to warm to room temperature without heating and was then heated to reflux overnight to generate **TRC6** (62% isolated yield) and **TRC7** (73% isolated yield, Figure 2).

**Figure 2:**
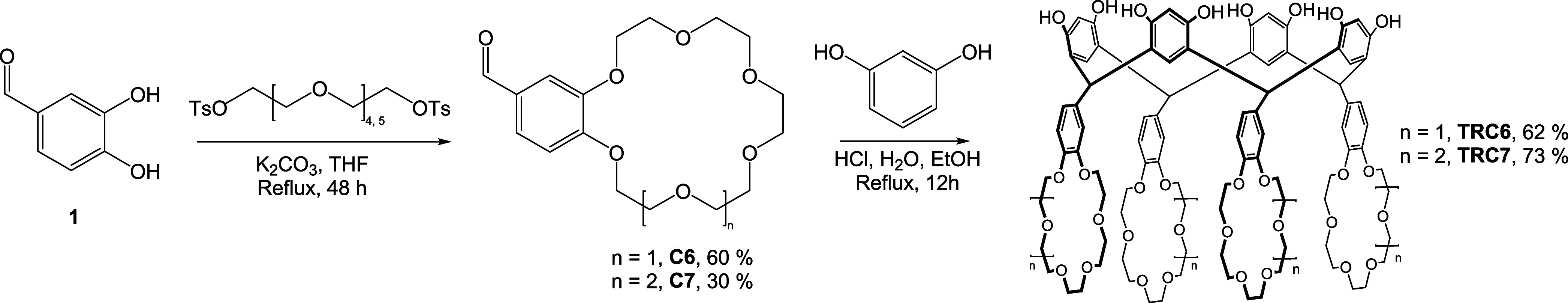
Synthesis of resorcinarene tetracrowns **TRC6** and **TRC7**.

### X-Ray Crystallography

In both **TRC6** and **TRC7**, the four crown ether moieties are in the axial positions at the methylene bridges and thus have the chair conformation (*rctt*). The chair conformation is the most common for resorcinarenes with bulky substituents at the methylene bridge.^33–35^ In the crystal structures (Figure 3), **TRC6** and **TRC7** reside on the center of inversion and have two independent crown ether moieties, which in turn have noticeably different conformations. The crown ether moiety conformations in **TRC6** and **TRC7** do not markedly deviate from similar uncomplexed DB18-C-6 ^36^ and DB-21-C-7 ^37^ molecule conformations. One of the crystallographically independent crown ether rings in both compounds is disordered (in a 50:50% ratio, Figure S12) with a markedly collapsed conformation, very likely due to the packing. The **TRC6** phenyl groups of the crown ethers were observed in an askew *anti*-arrangement (Figure 3, upper row), resulting in their respective crown ether moieties being misaligned, potentially hindering them from acting cooperatively toward larger cations that are too big to fit inside the ring. As such, if this conformation is indicative of solution-phase preferences, the crown ethers would only be capable of acting independently, both in an intra- and intermolecular manner, as demonstrated by the encapsulation of an opportunistic H_2_O solvate in **TRC6** (Figure 4a). However, in **TRC7** the crown ethers were observed in a *syn*-arrangement (Figure 3, lower row), such that pairs of them could act cooperatively, both in an intra- and intermolecular manner. Unfortunately, in the absence of coordinating cations, the crown ether rings have collapsed slightly, one more than the other, producing intermolecular pairs of crown ethers that have the potential to encapsulate guests in a polymeric manner along the crystallographic *c*-axis. We note that these analyses are of the solid-state structures of these materials. Their behavior in solution can be very different (solid-state forms are always the result of a compromise between the solution-phase preferred conformation of the molecule and the practical need for the compounds to form a single homogeneous phase in a tightly packed crystal lattice capable of being determined using X-ray crystallography. The solution-phase calculated forms predict that the crown-ethers can mutually support each other in binding guests (see Figure 9 below)

**Figure 3:**
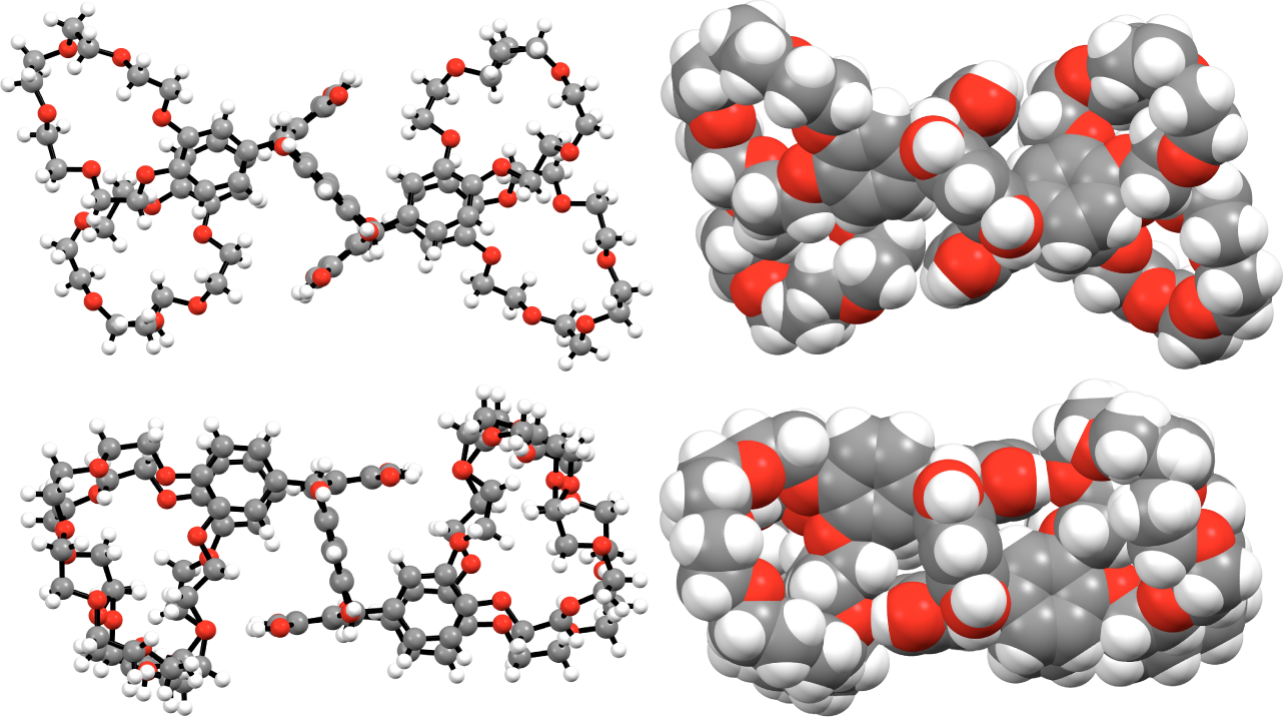
Ball-and-stick (*left*) and space-filling (*right*) figures of **TRC6** (*top*) and **TRC7** (*bottom*).

**Figure 4:**
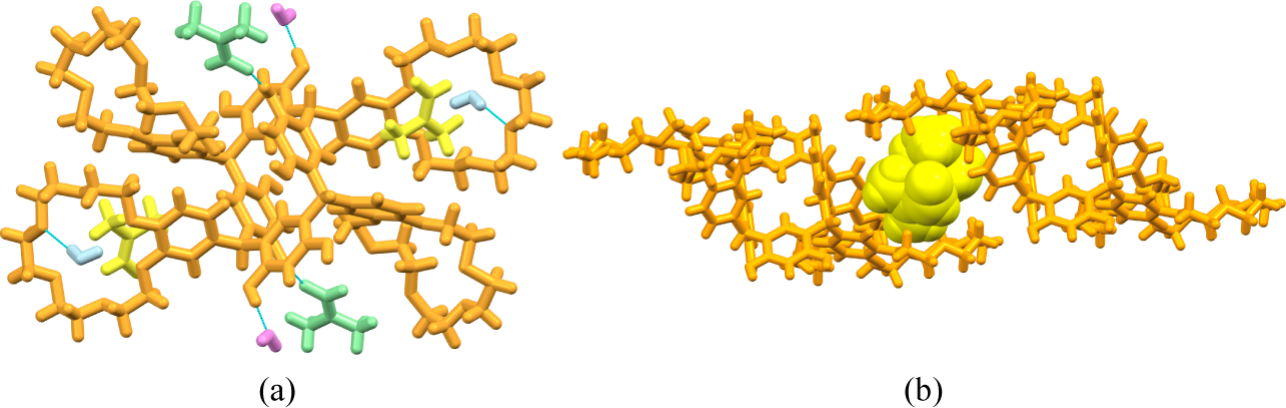
(a) The hydrogen-bonded DMF (green), the non-hydrogen-bonded DMF (yellow), the two hydrogen bonded water molecules (pink), and the water molecules encapsulated by the crown ether (light blue). (b) The DMF dimer (yellow) entrapped between two adjacent crown ethers of **TRC6** (other solvent molecules omitted for clarity; hydrogen bonds shown as turquoise dashed lines).

The crystallization solvents DMF and water could be located together with the **TRC6** in the lattice, giving an overall composition as **TRC6**·4­(DMF)·4­(H_2_O). Two of the DMF solvent molecules are hydrogen-bonded to the resorcinolic OH groups (Figure 4a), while the other, non-hydrogen-bonded, DMF molecules are entrapped as a dimer between two ether moieties from adjacent **TRC6** molecules (Figure 4b). Two of the crown ether moieties of **TRC6** bind a water molecule inside the crown ethers by hydrogen bonding (Figure 5a), these water molecules then join adjacent **TRC6** molecules as a 1-D chain (Figure 5b). The two other water molecules form a hydrogen-bonded dimer that is located in a small cavity and hydrogen-bonded to four molecules of **TRC6** (Figure 6).

**Figure 5:**
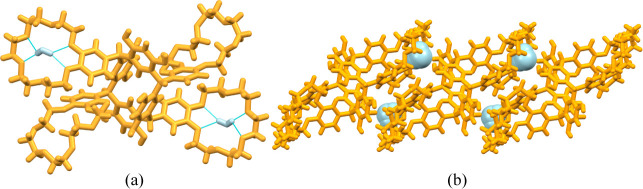
Crown ether hydrogen bonded (turquoise dashed lines) water molecules (a, light blue) and formation of a 1-D chain formed by hydrogen bonding of the encapsulated water to the crown ether (b) in **TRC6** (other solvent molecules omitted for clarity).

**Figure 6:**
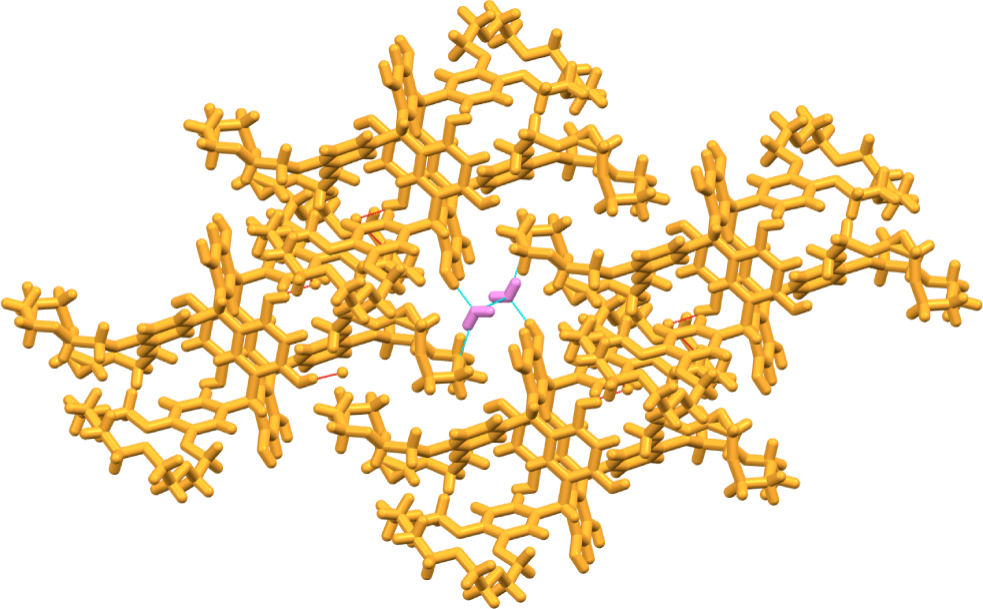
Two water molecules (pink) hydrogen bonded (turquoise dashed lines) to four molecules of **TRC6**.

However, the lattice of **TRC6** also has quite a large residual electron density that could not be modeled as either DMF or water molecules. This spurious electron density was removed by a SQUEEZE treatment which gave 138 electrons per unit cell as the undefined electron density.^38^ The removal of these 138 electrons results in a closed cavity of 449 Å^3^ (Figure S12) which is very likely filled with a mixture of highly disordered DMF and water molecules, *e.g*., 3­(DMF)·3­(H_2_O) or 2­(DMF)·6­(H_2_O).

The marked difference and explanation of the conformation of **TRC7** compared to **TRC6** are the four intermolecular hydrogen bonds from the same resorcinol OH group to the oxygen atoms of the crown ether oxygens (Figure 7). The O···O contact distances in the hydrogen bonds are 2.742(2) Å and 2.872(2) Å with the O–H···O angles of 169.8° and 175.4°, respectively. The Hirshfeld surface analysis^39^ (Figure S14) revealed some differences in the intermolecular H···O, H···C/π interactions, and H···H contacts. The nondisordered solvents molecules (DMSO and water) in **TRC6** led to a larger percentage of the H···O interactions (23.9%) when compared to **TRC7** (15.5%). Correspondingly, **TRC7** manifested a larger number of H···H contacts (72.0%) than **TRC6** (63.2%), while the number of H···C/π interactions are roughly the same, 11.8% (**TRC6**) and 12.0% (**TRC7**) (Figure S15).

**Figure 7:**
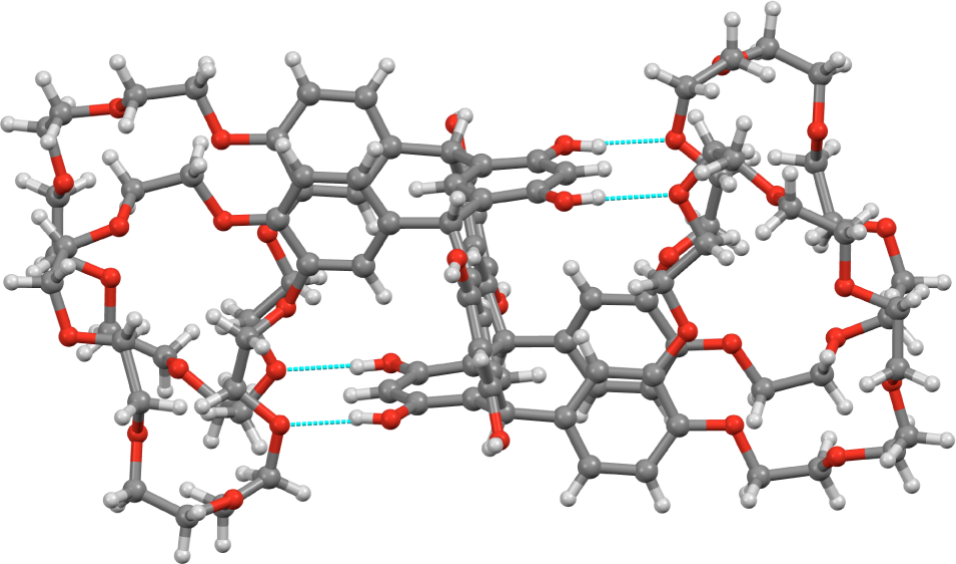
Four intramolecular hydrogen bonds (turquoise dashed lines) in **TRC7**.

The lattice of **TRC7** (crystallized from a DMF/H_2_O mixture) was found to exclusively contain heavily disordered solvent molecules, which were again removed using the SQUEEZE protocol. Removal of the spurious electron density revealed huge channels running along the *a*-axis occupying 26.6% of the unit cell volume (1583.6 Å^3^,Figure 8). The SQUEEZE protocol gave an estimate of the electron count for the unknown solvates present,^38^ with solvent accessible voids totaling 1560.5 Å^3^ with 428.7 electrons per unit cell. Based on the electron count eight DMF and 11 H_2_O molecules could occupy the channels.

**Figure 8:**
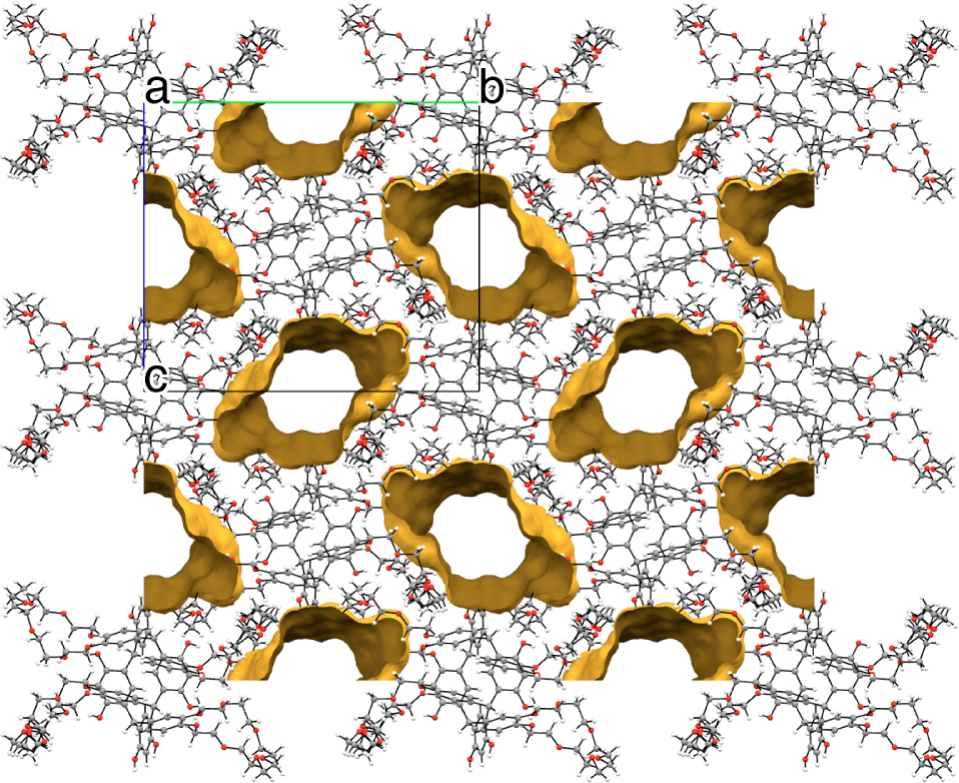
Channels (highlighted with orange boundaries) running through the lattice of **TRC7** along the crystallographic *a*-axis.

### Computation

The conformational sampling of the **TRC6** and **TRC7** resorcinarene macrocycles in an aqueous environment revealed significant insights into their structural preferences (Figure 9). The minimum energy conformation of **TRC6** (Figure 9a) adopts a characteristic asymmetric arrangement with one crown ether ring oriented upward while the other three crown ether rings point downward in a “three-down, one-up” configuration. This asymmetric orientation is noteworthy as it suggests a preferred conformational state that balances various intramolecular forces.

**Figure 9:**
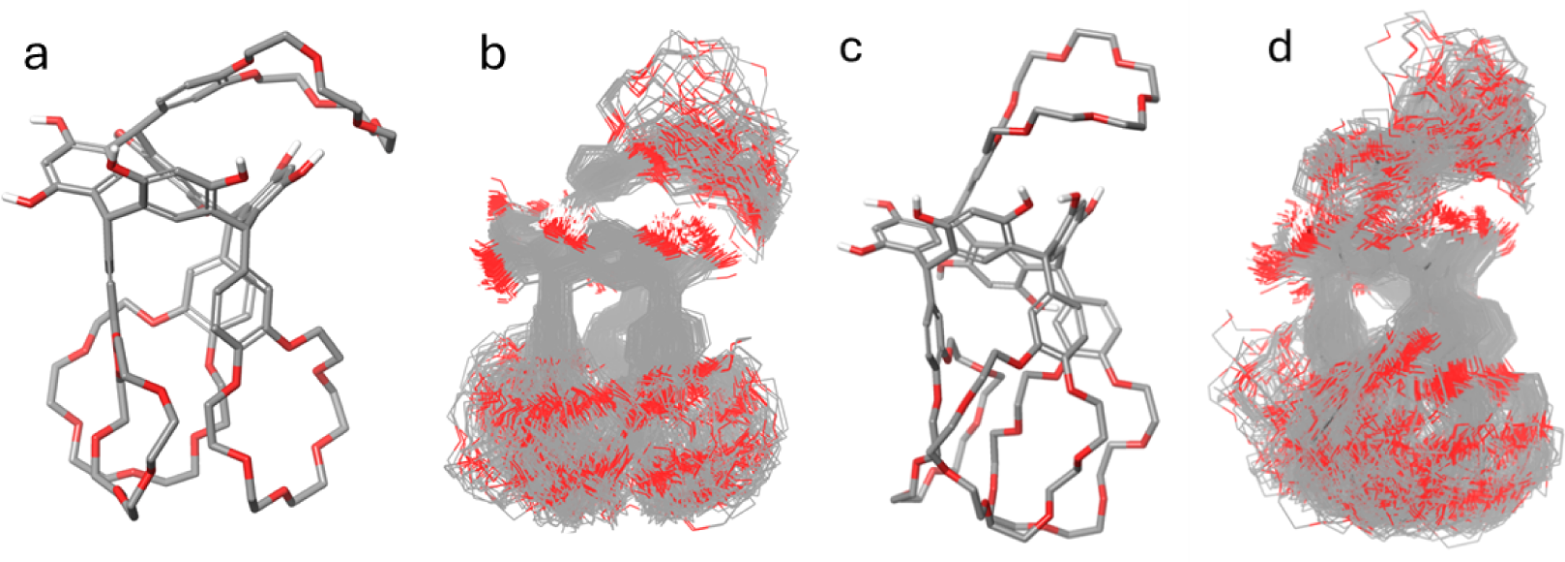
Conformational analysis of resorcinarene macrocycles **TRC6** and **TRC7**. (a) Minimum energy conformation of **TRC6** displaying the characteristic “three-down, one-up” arrangement of crown ether rings. (b) Overlay of all generated conformers for **TRC6** showing flexibility in the crown ether regions while maintaining core structural integrity. (c) Minimum energy conformation of **TRC7** exhibiting similar “three-down, one-up” orientation. (d) Conformational ensemble of **TRC7** showing comparable flexibility patterns in the crown ether moieties while preserving the central cavity structure. In all panels, gray represents carbon atoms, red represents oxygen atoms, and white represents hydrogen atoms.

The superimposition of all generated conformers (Figure 9b) demonstrates the considerable conformational flexibility of the **TRC6** macrocycle, particularly in the crown ether regions. The oxygen atoms (shown in red) exhibit high mobility throughout the conformational ensemble, indicating that these regions can adapt to different environments. Despite this flexibility, the core resorcinarene scaffold maintains a relatively consistent shape across the conformational ensemble, with the central cavity largely preserved. Similar conformational preferences were observed for **TRC7**, which also favored the “three-down, one-up” arrangement of crown ether rings in its minimum energy structure. This consistency between **TRC6** and **TRC7** suggests that this conformational motif represents a fundamental energetic preference for these crown ether-bridged resorcinarene systems, likely driven by a balance of factors including steric effects, and potential intramolecular hydrogen bonding. The preservation of the central cavity across the conformational ensemble for both macrocycles is particularly significant from a host–guest chemistry perspective, as it suggests these molecules could function as stable receptors despite their inherent flexibility. The conformational flexibility observed primarily in the crown ether linking regions rather than the core resorcinarene structure implies that these molecules might accommodate various guest molecules through induced-fit mechanisms while maintaining their overall binding pocket geometry. The specific packing observed in the solid state may be more a feature of a preferred, denser, conformation facilitating crystallization rather than an inherent preference of the molecule when diluted in solution.

### Complexation Studies

#### NMR Spectroscopy

NMR titrations were performed to qualitatively assess possible binding interactions between the resorcinarene crowns and alkali metals K^+^ and Rb^+^. Results from NMR measurements in DMSO indicate that in solution, the complexes are in rapid equilibrium with the free components, culminating in a single set of signals as observed in the NMR spectra of the mixtures. Binding of the guests can be determined by monitoring the shielding or deshielding effects of the signals of either the host or guest.^40–42^ The binding of K^+^ to **TRC6** led to deshielding of the receptor’s ArH protons at 6.5 ppm at different equivalents of the guest (Figure 10a). Shielding (∼5.9 ppm) and deshielding (∼5.6 ppm) of the −CH signals indicate a change in the electronic environment upon binding the guest through cation-dipole interaction. Similar changes but more significant shielding were observed when Rb^+^ binds to **TRC6** (Figure S17). The larger Rb^+^ cation has a greater effect on the electronic environment of the receptors when bound.

**Figure 10:**
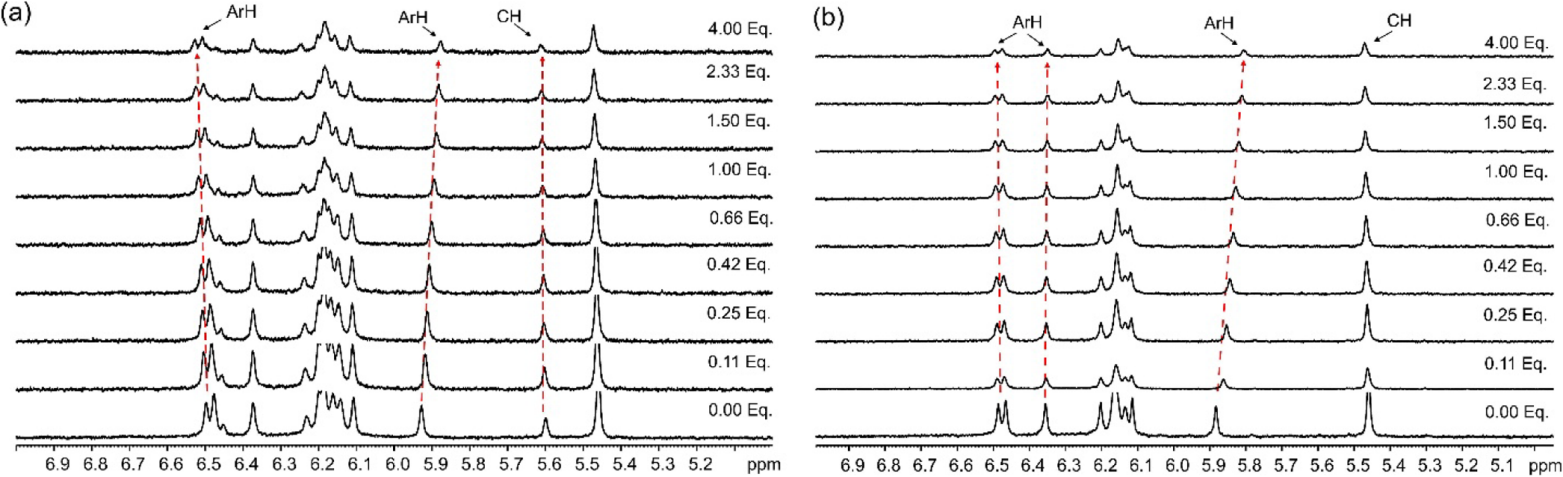
Stacked ^1^H NMR spectra (DMSO-D_6_, 298 K) showing (a) pure **TRC6** and up to four equivalents of KCl, (b) pure **TRC7** and up to four equivalents of RbCl. The dashed lines indicate the signal changes in ppm.

Similarly, binding of K^+^ to **TRC7** resulted in deshielding of the receptor’s ArH proton at 6.3–6.6 ppm (Figure S19). Likewise, changes in other ArH signals around 5.9 ppm indicate interaction with the crown ethers. Very small changes of the −OCH_2_– signals between 3.0 and 4.0 ppm were also observed. These interactions all confirm that **TRC7** also binds K^+^. Similar binding characteristics were observed for both **TRC6** and **TRC7** in the presence of the slightly larger Rb^+^ with deshielding of the aromatic signals 6.3–6.6 ppm and shielding of the signals around 5.9 ppm (Figures 10b and S17). Job plots revealed a 1:1 binding stoichiometry between the metal ions (K^+^ and Rb^+^) and **TRC6**, and 1:2 stoichiometry with **TRC7** in the highly competitive DMSO (Figure S26). The competitive nature of the competing DMSO solvent could explain the lack of higher binding stoichiometry for these species in solution.

Considering the competitive nature of DMSO as a solvent, we tested the binding of the cations in acetonitrile. Due to the insolubility of the Cl^–^ salts in acetonitrile, only the BF_4_^–^ salts were tested. Up to five equivalents of the cations were added to the receptors. The binding of K^+^ to **TRC6** led to deshielding of the receptor’s ArH protons at 6.5 ppm at different equivalents of the guest (Figure 11a). Shielding of the ArH signals around 6.1 ppm was also observed. Interestingly, the −CH signal around 5.5 ppm splits into two after up to 3 equiv of the K^+^ cation, suggesting a potential slow binding process. Similar changes were observed with **TRC7** (Figure 11b). However, no splitting of the −CH signal around 5.5 ppm was observed. This could indicate that the binding by the larger crown-7 is a much faster process on the NMR time scale.

**Figure 11:**
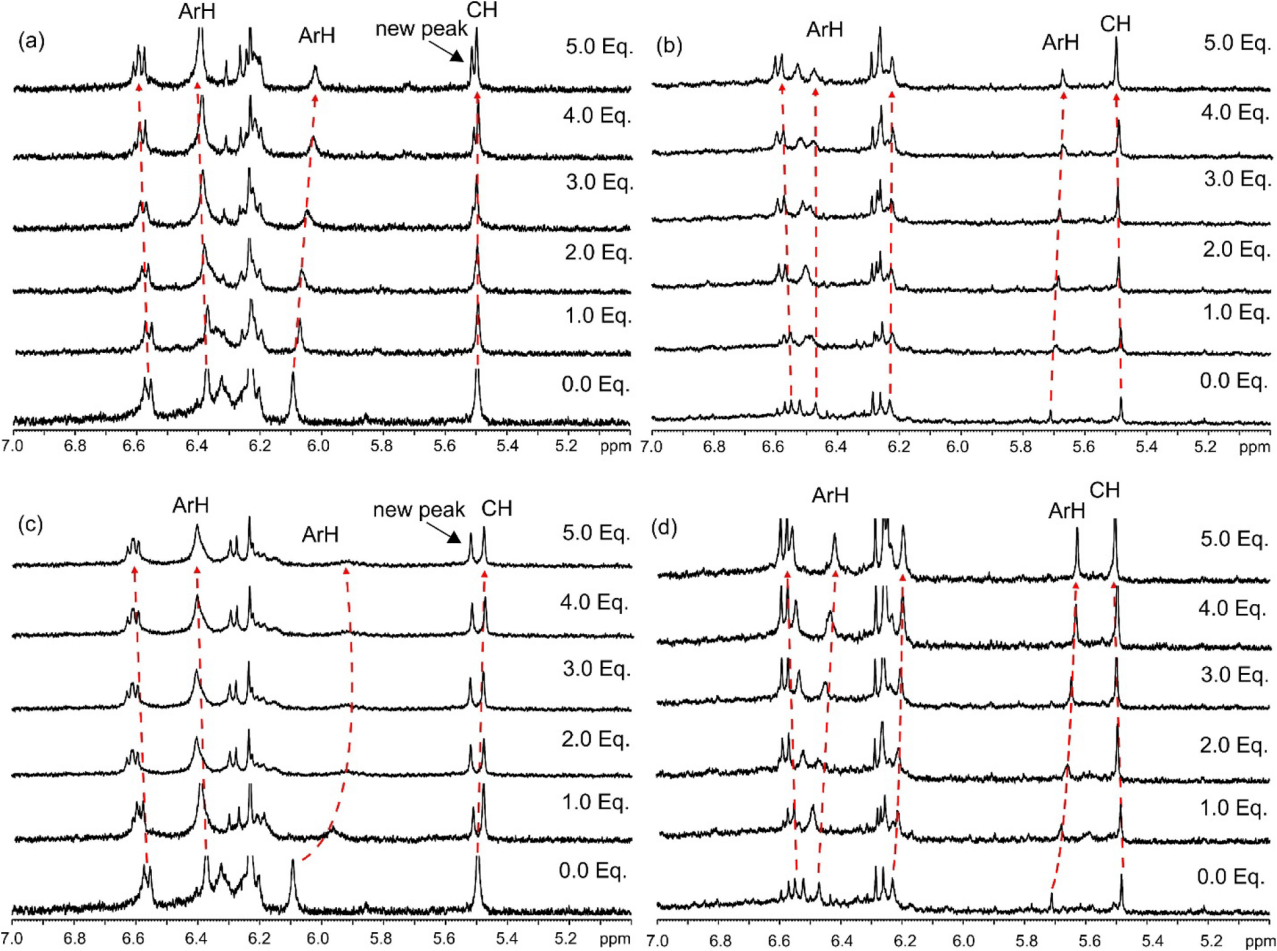
Stacked ^1^H NMR spectra (CD_3_CN, 298 K) show up to four equivalents of (a) KBF_4_ into **TRC6**, (b) KBF_4_ into **TRC7**, (c) RbBF_4_ into **TRC6**, and (d) RbBF_4_ into **TRC7**. The dashed lines indicate the signal changes in ppm.

The binding of RbBF_4_ salt by the receptors was also tested in acetonitrile. Up to five equivalents of the cations were added to the receptors. The binding of Rb^+^ to **TRC6** led to deshielding of the receptor’s ArH protons at 6.5 ppm at different equivalents of the guest (Figure 11c). Substantial shielding of the ArH (∼0.21 ppm) signals around 6.1 ppm was also observed. It was observed that the splitting of the −CH signal around 5.5 ppm started at 1 equiv of the Rb^+^ cation, suggesting a slow binding process of the larger cation. Similar changes were observed with TRC7 with no splitting of −CH signal around 5.5 ppm (Figure 11d). As indicated above, the binding by the larger crown-7 is a much faster process in the NMR time scale.

#### Isothermal Calorimetry Titration (ITC)

We employed isothermal calorimetry titration (ITC) to quantify the binding processes and get an insight into the thermodynamics of the binding. The thermodynamics of host–guest complexation were assessed using a series of ITC experiments in acetonitrile (Figures S27–S30 and Table 1). For solubility reasons, only the BF_4_^–^ salts were used. The data could not be fitted to one-site or two-site binding models. As such we fitted the ITC data to a sequential binding model to obtain the binding constants (*K*_a_), Δ*H*, Δ*S*, and Δ*G*. We observed up to three sequential binding events between **TRC6** and KBF_4_, and only two binding events for **TRC6** and RbBF_4_, and for both binding with **TRC7**. Complex formation between **TRC6** and each of KBF_4_ and RbBF_4_ had negative Δ*H* and Δ*G* values, signifying exothermic complexation spontaneous at all temperatures. However, the first binding interaction between **TRC6** and KBF_4_ produced an endothermic reaction but with a comparably low level of entropy. On the other hand, the negative TΔ*S,* ΔG and positive Δ*H* values involved in complexes between **TRC7** and each of KBF_4_ and RbBF_4_ indicate endothermic complexation that is spontaneous only at high temperatures with increased entropy. Comparatively, **TRC6** with a smaller cavity size binds the cations tighter than **TRC7**, which can be attributed to a better size match for K^+^ and Rb^+^ compared to **TRC7.**

**Table 1:** Thermodynamic Binding Parameters of Formed Complexes Between the **TRC6**, **TRC7** as Hosts, and KBF_4_, RbBF_4_ as Guests by ITC^a^

Complex	*K*_a_ (M^–1^)	Δ*H* 10^3^ × kJ/mol	*T*Δ*S* 10^3^ × kJ/mol	Δ*G* kJ/mol
**KBF**_**4**_ + **TRC6**	*K*_1_ = 1.483 × 10^4^	Δ*H*_1_ = 0.57	*T*Δ*S*_1_ = 0.59	Δ*G*_1_ = −23.51
	*K*_2_ = 1.458 × 10^2^	Δ*H*_2_ = −4.23	*T*Δ*S*_2_ = −4.21	Δ*G*_2_ = −14.26
	*K*_3_ = 2.501 × 10^1^	Δ*H*_3_ = −2.50	*T*Δ*S*_3_ = −2.49	Δ*G*_3_ = −9.32
**RbBF**_**4**_ + **TRC6**	*K*_1_ = 5.423 × 10^1^	Δ*H*_1_ = −5.25	*T*Δ*S*_1_ = −5.24	Δ*G*_1_ = −11.14
	*K*_2_ = 1.267 × 10^3^	Δ*H*_2_ = −5.00	*T*Δ*S*_2_ = 5.02	Δ*G*_2_ = −15.34
**KBF**_**4**_ + **TRC7**	*K*_1_ = 3.585 × 10^1^	Δ*H*_1_ = 10.00	*T*Δ*S*_1_ = 10.01	Δ*G*_1_ = −3.86
	*K*_2_ = 7.943 × 10^1^	Δ*H*_2_ = 5.00	*T*Δ*S*_2_ = 5.01	Δ*G*_2_ = −9.38
**RbBF**_**4**_ + **TRC7**	*K*_1_ = 1.306 × 10^1^	Δ*H*_1_ = 9.99	*T*Δ*S*_1_ = 9.99	Δ*G*_1_ = −0.96
	*K*_2_ = 3.808 × 10^2^	Δ*H*_2_ = 5.00	*T*Δ*S*_2_ = 5.01	Δ*G*_2_ = −12.36

a ITC done at 298 K in CH_3_CN.

## Conclusions

We have successfully synthesized two resorcinarene-crown ethers, **TRC6** and **TRC7**, revealing their conformational preferences in the solid state. Single crystal analysis of **TRC6** demonstrated its ability to accommodate guests via the crown ether substituents in multiple ways, whereas **TRC7** displayed continuous channels in the solid state that might facilitate selective guest uptake within its structure. Hirshfeld surface analysis revealed some differences in the intermolecular H···O, H···C/π interactions, and H···H contacts between the **TRC6** and **TRC7**. Computational results suggest the packing observed in the solid state may be a solid-state feature rather than an inherent preference of the molecule when in solution. Host–guest binding studies revealed 1:1 or 1:2 complexes with K^+^ and Rb^+^ cations in highly competitive DMSO. In acetonitrile more intense signal changes are observed. Splitting of the **TRC6** −CH signal suggests a slow binding process that starts at three equivalents of the smaller K^+^ cations and at one equivalent of the larger Rb^+^ cation. Quantification of the binding process was done through isothermal calorimetric titration studies via a sequential binding model. The results suggest that the **TRC6** with the smaller crown binds to the cations more tightly than the larger **TRC7**. These findings highlight the structural versatility and binding capabilities of the resorcinarene-crown ethers and their potential applications in supramolecular chemistry and ion recognition.

## Experimental Section

The solvents used for synthesis, ^1^H NMR spectroscopy and crystallization experiments were reagent grade and were used as received without further purification. Other chemicals were purchased from Sigma-Aldrich, AK Scientific, Oakwood Chemicals, Alfa Aesar or Acros Chemicals and were used without further purification unless otherwise noted.

### Synthesis of Lower Rim Resorcinarenes


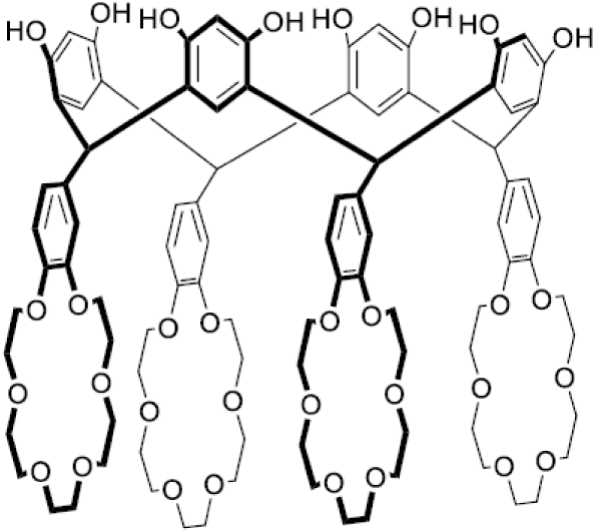
 **TRC6:** To a solution of resorcinol (0.04 g, 0.36 mmol) in ethanol (0.9 mL), water (0.9 mL) and hydrochloric acid (conc., 0.45 mL), 4’-formylbenzo­[18C6] (0.125 g, 0.36 mmol) in ethanol (0.45 mL) was added dropwise at 0–5 °C under nitrogen. After addition of the aldehyde, the mixture was left to warm to room temperature without heating and was then heated at reflux overnight.^43^ The reaction was cooled to room temperature, the precipitate was separated, washed with ethanol and H_2_O and dried under vacuum to give the product as a pinkish-orange solid (0.39 g, 0.22 mmol, 62% Yield). R**_f_**: 0.19 in 6:4 EtOAc:MeOH. ^**1**^ **H NMR** (300 MHz, DMSO*-d6*) δ 8.40 (d, *J* = 2.8 Hz, 8H), 6.48 (d, *J* = 8.1 Hz, 4H), 6.36 (s, 2H), 6.21–6.08 (m, 12H), 5.92 (s, 2H), 5.45 (s, 4H), 3.80–3.50 (m, 80H). ^**13**^ **C NMR** (76 MHz, *CDCl*_3_) δ 151.7, 151.5, 146.6, 146.5, 144.8, 144.7, 136.2, 121.0, 120.4, 119.7, 113.7, 111.9, 69.1, 69.1, 69.0, 68.4, 68.2, 67.7, 67.0, 40.9. **HR-ESI-MS** *m*/*z* calcd for C_92_H_112_O_32_ [M + H]^+^: 1729.7215, found: 1729.7134 and calculated for [M + 2H]^2+^: 865.3644, found: 865.3641.


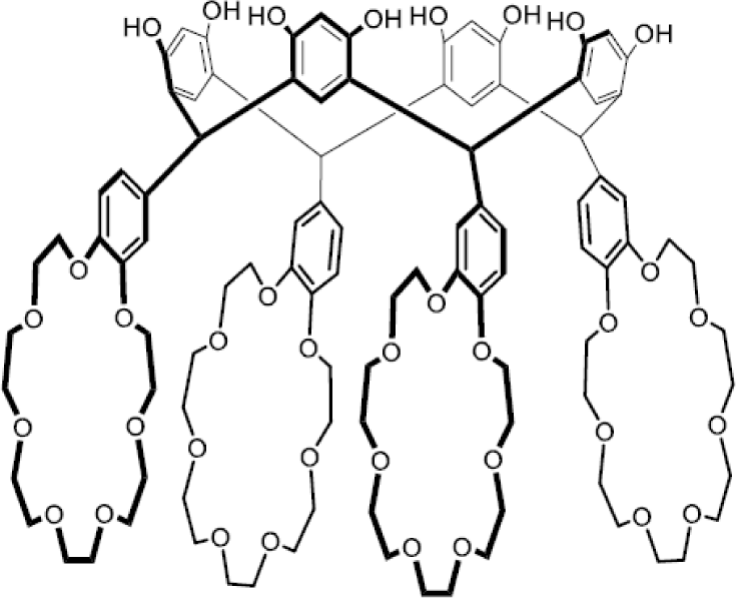
 **TRC7:** To a solution of resorcinol (0.085 g, 0.77 mmol) in ethanol (1.91 mL), water (1.91 mL) and hydrochloric acid (conc., 0.95 mL), 4’-formylbenzo­[21C7] (0.3 g, 0.78 mmol) in ethanol (0.95 mL) was added dropwise at 0–5 °C under nitrogen. After addition of the aldehyde, the mixture was left to warm to room temperature without heating and was then heated at reflux overnight. The reaction was cooled to room temperature, the precipitate was separated, washed with ethanol and H_2_O and dried under vacuum to give the product as a white solid. (1.07 g, 0.56 mmol, 73% yield). **R**_**f**_: 0.32 in 6:4 EtOAc:MeOH. ^**1**^ **H NMR** (300 MHz, DMSO) δ 8.39 (d, J = 7.1 Hz, 8H), 6.46 (d, *J* = 8.2 Hz, 4H), 6.34 (s, 2H), 6.22–6.07 (m, 12H), 5.87 (s, 2H), 5.44 (s, 4H), 3.80–3.47 (m, 96H). ^**13**^ **C NMR** (76 MHz, CDCl3) δ 152.6, 152.4, 147.2, 145.5, 137.1, 121.7, 121.2, 120.6, 114.5, 112.7, 70.4, 70.4, 70.3, 70.2, 70.0, 69.9, 69.4, 69.1, 68.5, 67.9, 41.7. **HR-ESI-MS** *m*/*z* calcd for C_100_H_128_O_36_ [M + H]^+^: 1906.8297, found: 1906.8185.

#### Computational Methodology

Conformational sampling of resorcinarene molecules **TRC6** and **TRC7** was performed using Schrödinger’s Macrocycle Conformational Sampling tool.^44^ The initial geometries were obtained from the crystal structures. The OPLS4 force field^45^ was employed for energy calculations, with electrostatic interactions modeled using the GB/SA water implicit solvent model. Conformational search parameters were optimized for thorough exploration of the macrocyclic conformational space. The simulation was set to run for 5000 cycles, with 5000 LLMOD (Large-scale Low-Mode) search steps. Redundant conformers were eliminated using a root-mean-square deviation (RMSD) cutoff of 0.75 Å. An energy window of 10.0 kcal/mol was applied for saving structures, ensuring that only energetically reasonable conformers were retained. Enhanced torsion sampling options were selected to improve the exploration of relevant torsional space for these highly flexible macrocycles. Eigenvectors were determined for each new global minimum to efficiently direct the conformational search. This approach allows for a comprehensive mapping of the conformational landscape of both **TRC6** and **TRC7** resorcinarene macrocycles, providing insight into their structural preferences and energetically favorable conformations.

#### X-Ray Crystallography

Single crystals were obtained from slow evaporation from untreated DMF. The single crystal X-ray data for **TRC6** and **TRC7** were collected using an Agilent SuperNova dual-source diffractometer equipped with an Atlas detector using mirror-monochromatic Cu-*K*α (λ = 1.54184 Å) radiation. The structures were solved by intrinsic phasing (SHELXT)^46^ and refined by full-matrix least-squares on *F* ^2^ using Olex2,^47^ utilizing the SHELXL module.^48^ Anisotropic displacement parameters were assigned to non-H atoms and isotropic displacement parameters for all H atoms were constrained to multiples of the equivalent displacement parameters of their respective parent atoms, with *U*_iso_(H) = 1.2*U*_eq_(C) for CH/CH_2_ groups and *U*_iso_(H) = 1.5*U*_eq_(C) for CH_3_/OH groups. The main details of crystal data collection and refinement parameters are presented in Table S1.

#### NMR Spectroscopy

The ^1^H NMR spectra were recorded on various Bruker NMR spectrometers: Bruker Avance DRX 400, Bruker Avance DPX 300 Ultra Shield, and Bruker Avance III 500 MHz. The NMR chemical shifts (δ) are reported in ppm and are calibrated against residual solvent signals of CDCl_3_ (δ 7.26), DMSO-*d*_6_ (δ 2.50) or CD_3_CN (δ 1.94). Further details of all protocols of the precursor compounds, NMR spectra, and other characterization/methodology details can be found in the SI. Job Plot and NMR complexation studies were done in the respective deuterated solvents. For the titrations, calculated volumes of the guest were titrated into tetracrown solution to achieve up to 5 equiv. NMR measurements were done on a 400 MHz Avance III Bruker NMR instrument.

#### Isothermal Calorimetry Titration

The ITC experiment was carried out by filling the sample cell with one sample (substrate, 1 mM), filling the syringe with the second sample (titrant, 10 mM), and titrating via computer-automated injector at 298 K. Blank titrations into plain solvent were also performed and subtracted from the corresponding titration to remove any effect from the heats of dilution from the titrant. Heat changes from ITC titrations were recorded using NanoITC instrument from TA Instruments at 298 K. Isotherms (available in Supporting Information) and thermodynamic parameters from a sequential fitting model (*K*_a_, Δ*H and* Δ*S*) were obtained using the NaNoAnalyze software. Gibbs’ free energy Δ*G* was subsequently calculated at 298 K and recorded (Table 1). ITC experiments were not performed in DMSO due to the high heat of dilution associated with DMSO.

## Supplementary Material



## Notes

Synthesis or precursors, X-ray experimental details, ^1^H and ^13^C NMR spectra, and Job’s plot details are included in the Supporting Information (PDF)
