# Supplementary material for: Synthesis and X‑ray Structures of Bis-Functional Resorcinarene Crown Ethers
Source: Cryst Growth Des. 2025 Jun 25;25(14):5427–35. doi: 10.1021/acs.cgd.5c00295 (PMC12272548; doi:10.1021/acs.cgd.5c00295)
Supplement: Supplementary file 1 [file cg5c00295_si_001.pdf]

Synthesis and X-Ray Structures of Bis-Functional Resorcinarene Crown Ethers

Frank Boateng Osei,[a] Sanaz Nadimi,[b] Jas S. Ward,[c] Sarah Nasri,[b] Abd Al-Aziz A. Abu-Saleh,[b,d] Elham Pourian,[b] John F. Trant,[b,d,e,f] Kari Rissanen,[c] Ngong Kodiah Beyeh[a*]

[a] *Oakland University, Department of Chemistry, 146 Library Drive, Rochester, Michigan, 48309-4479, USA*

[b] *University of Windsor, Department of Chemistry and Biochemistry, 401 Sunset Avenue, Windsor, ON, N9B 3P4 Canada*

[c] *University of Jyvaskyla, Department of Chemistry, Survontie 9 B, FI-40014 Jyväskylä, Finland*

[d] *Binary Star Research Services, LaSalle ON, N9J 3X8, Canada*

[e] *WE-Spark Research Institute, 401 Sunset Ave., Windsor, ON, N9B 3P4, Canada*

[f] *Department of Biomedical Sciences, University of Windsor, 401 Sunset Ave., Windsor, ON, N9B 3P4, Canada*

Table of Contents

I. Materials and general synthetic procedure 2
II. Synthesis 3
III. Solution NMR 6
IV. X-Ray crystallography Details 11
V. Complexation Studies 13
VI. References 24

I. Materials and general synthetic procedure

All the reagents and solvents used for synthesis, crystal growth, and NMR experiments were purchased from commercial sources (Sigma Aldrich) and were used as received. Other chemicals were purchased from Sigma-Aldrich, AK Scientific, Oakwood Chemicals, Alfa Aesar or Acros Chemicals and were used without further purification unless otherwise noted. All heated reactions were conducted using a silicone oil bath on IKA RET Basic stir plates equipped with a P1000 temperature probe. All reactions under elevated temperatures used all glass joints in contact with the solvent. Septa were generally used at the top of condensers and for reactions not under elevated temperatures. Vacuum and gases were introduced from a Schlenk line using needles through septa during reactions, and generally directly using glass adapters during drying. Thin layer chromatography was performed to track synthetic progress by using EMD aluminum-backed silica 60 F254-coated plates and were visualized using either UV-light (254 nm), $KMnO_4$, vanillin, Hanessian's stain, Dragendorff or phosphomolybdic Acid (PMA)'s stain. Standard work-up procedure for all reactions undergoing an aqueous wash, unless otherwise stated, involved back extraction of every aqueous phase, a drying of the combined organic phases with anhydrous magnesium sulphate, filtration either using vacuum and a sintered-glass frit or through a glass-wool plug using gravity, and concentration under reduced pressure on a rotary evaporator (Büchi or Synthware). Depending on the compound, various methods were used to purify titled compounds. The exact conditions used for purification would be mentioned under each synthetic procedure. Low Resolution Mass Spectrometry was carried out by an Advion Expression-LCMS with an Electro Spray Interface (ESI) and was operated on positive mode. The conditions for the acquisition parameters were: capillary voltage 160 V and temperature 250 °C; source gas temperature 250 °C at a flow of 3 L × min^{-1} and a ESI Voltage of 3.5 kV. The scan range was from 100-1000 m/z. The data was analyzed on Advion Dataexpress. HRMS were sent out to external facilities (Brock University, Ontario, Canada and Queens University, Ontario, Canada) to do ESI, positive mode on Bruker HCT Plus ion-trap mass spectrometer and an Agilent AdvanceBio 6545XT LC/QTOF mass spectrometer, respectively.

II. Synthesis

The resorcinarene backbone was synthesized according to general standard procedures (Figure S1**)**. Briefly, polyethylene glycol was doubly tosylated in high yield. Combining the tosyl glycols and benzaldehyde **1**, crown ethers **C6** and **C7** were produced. Finally, the resorcinarenes were synthesized by an acid catalyzed condensation reaction between resorcinol and the aldehyde-functionalized benzo crown ethers[1]. The resulting product was precipitated in water, filtered, and washed with water until neutral and dried with vacuum. This provided clean samples of the desired product; none of the undesirable oligomers remained with the solid.

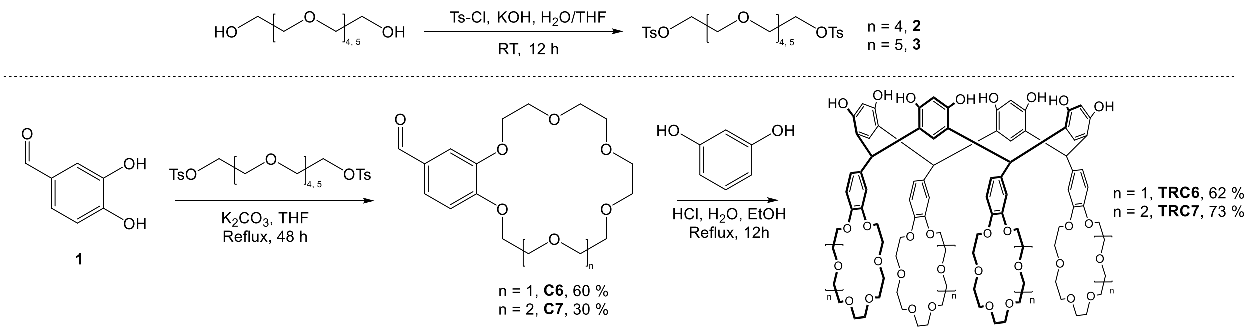

Figure S1. Synthesis of Resorcinarene tetracrowns 6 and 7 (**TRC6** and **TRC7**).

General Method A. Ditosylation of PEG components

The synthesis followed procedures based on previous literature.[2] The oligo ethylene glycol (42 mmol) and 4-toluenesulfonyl chloride (24.1 g, 126 mmol) were dissolved in THF (110 mL) and cooled down to 0 °C in an ice bath. No special precautions were taken with respect to the atmosphere or the presence of water. KOH (14 g, 252 mmol), predissolved in H_2O (16 mL) and allowed to cool to ambient, was slowly added into the reaction mixture. The reaction mixture was then allowed to slowly warm up to room temperature and stirred overnight. Upon completion, THF was evaporated, and H_2O (30 mL) was added followed by an extraction with diethyl ether (3 × 30 mL). The combined organic layers were washed with brine, dried with $MgSO_4$ and the solvent removed under reduced pressure. The resulting ditosylated compounds were purified through flash chromatography using 1:1:4 DCM:Acetone:Hexanes to elute excess Ts-Cl, followed by a methanol flush to obtain the product. Note to the reader: unreacted oligoethylene glycol, or mono-tosylated material was almost completely removed in the aqueous wash, they have very poor solubility in diethyl ether.

3,6,9,12-tetraoxatetradecane-1,14-diyl bis(4-methylbenzenesulfonate) (2)

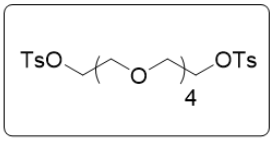

Following general method A with penta-ethyelene glycol as the PEG reagent, the product is a white solid. The yields for the reaction ranged from 77% to 90% over 3 separate reactions. R_f: 0.52 in 2 Hexanes: 1 DCM: 1 Acetone . m.p.: 43-45 ℃. **^{1}H NMR** (300 MHz, *$CDCl_3$*) δ 7.82 – 7.73 (m, 4H), 7.32 (d, J = 8.1 Hz, 4H), 4.18 – 4.09 (m, 4H), 3.71 – 3.62 (m, 4H), 3.57 (d, J = 6.6 Hz, 12H), 2.43 (s, 6H). **HRMS (ESI)** m/z calcd for $C_{24}H_{35}O_{10}S_2^+$ [M+H]$^+$: 547.1666. Found: 547.1665; m/z calcd for $C_{24}H_{34}O_{10}S_2Na^+$ [M+Na]$^+$: 569.1486. Found: 569.1489. Data is consistent with the literature results. [3]

3,6,9,12,15-pentaoxaheptadecane-1,17-diyl bis(4-methylbenzenesulfonate) (3)

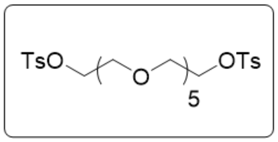

Following general method A with hexaethylene glycol as the PEG reagent, the product is a thick slightly opaque off-white oil. The yields for the reaction ranged from 93-95% over 2 reactions. R_f: 0.43 in 2 Hexanes: 1 DCM: 1 Acetone. **^{1}H NMR** (300 MHz, $CDCl_3$) δ 7.78 (d, *J* = 8.4 Hz, 4H), 7.44 – 7.27 (m, 4H), 4.25 – 4.07 (m, 4H), 3.77 – 3.64 (m, 4H), 3.64 – 3.48 (m, 16H), 2.44 (d, *J* = 3.6 Hz, 6H). **^{13}C NMR** (76 MHz, $CDCl_3$) δ 144.9, 133.1, 129.9, 128., 70.8, 70.7, 70.65, 70.60, 69.4, 68.8, 21.8. **HRMS (ESI)** m/z calcd for $C_{26}H_{39}O_{11}S_2^+$ [M+H]$^+$: 591.1928. Found: 591.1927; m/z calcd for $C_{26}H_{38}O_{11}S_2Na^+$ [M+Na]$^+$: 613.1748. Found: 613.1752; m/z calcd for $C_{26}H_{38}O_{11}S_2K^+$ [M+K]$^+$: 629.1487. Found: 629.1487.

General Method B. Synthesis of crown ethers

Following literature protocols,[4] 3,4-dihydroxybenzaldehyde **1** (2.47g, 18.3 mmol) and carbonate salt (54.8 mmol) was dissolved and diluted in acetonitrile or THF (200 mL) and heated to reflux in a two-neck flask with a magnetic stir bar under an argon atmosphere. Tosylated-PEG (**2** or **3**, 18.3 mmol) was dissolved in 50 mL acetonitrile or THF in a syringe and added dropwise into the reaction flask over 1 hour. The reaction was then maintained at reflux for 48 hours, then cooled to ambient, and the contents of the round bottom flask were concentrated in *vacuo*. The crude residue was resuspended in CH_2Cl_2 (20 mL) and washed with water (10 mL) then brine (10 mL). The organic layer was dried with magnesium sulfate, filtered and concentrated *in vacuo*. Finally, the concentrated material was washed with hot diethyl ether and the ether was decanted from the oily mixture (3 × 25 mL). The ether collection was combined and stored over left in the freezer overnight to produce a white solid crop of crown ether solids.

4'-Formylbenzo[18C6] (C6)

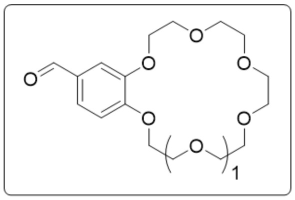

Yield: 60%. Potassium carbonate was used as base in General Method B. Title compound is a fluffy white solid. R_f = 0.41 (Hexanes: CH_2Cl_2:Acetone 2:1:1). **^{1}H NMR** (300 MHz, *$CDCl_3$*) δ 9.83 (s, 1H), 7.51 – 7.36 (m, 2H), 6.95 (d, *J* = 8.2 Hz, 1H), 4.23 (q, *J* = 5.3 Hz, 4H), 3.95 (dt, *J* = 9.2, 4.5 Hz, 4H), 3.83 – 3.75 (m, 4H), 3.75 – 3.66 (m, 8H). **^{13}C NMR** (126 MHz, *$CDCl_3$*) δ 190.8, 130.0, 129.78, 127.88, 126.7, 111.90, 111.1 70.89, 70.86, 70.70, 70.62, 70.56, 70.48, 70.39, 69.2, 69.12, 68.89, 68.86, 68.55. **HRMS (EI)** m/z calcd for $C_{17}H_{25}O_7^+$ $[M+H]^+$: 341.1565. Found: 341.1557, **LC-LRMS** Found: 341.9 ($M+H^+$), 359.9 ($M+NH_4^+$), 363.6 ($M+Na^+$). **IR (solid)/cm^{-1}**: ṽ= 3580, 3481, 2938, 2867, 2219 (C-H aldehyde), 1684 (C=O aldehyde), 1584, 1260, 1113.

4'-Formylbenzo[21C7] (C7)

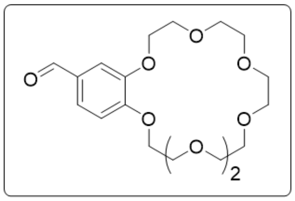

Yield: 40%. Cesium carbonate was used as base in General Method B. Compound can be a fluffy white solid or a very thick yellow oil upon settling. R_f = 0.38 in 2 Hexane: 1 CH_2Cl_2: 1 Acetone. **^{1}H NMR** (500 MHz, DMSO) δ 9.83 (s, 1H), 7.59 – 7.48 (m, 1H), 7.39 (d, J = 1.9 Hz, 1H), 7.18 (d, J = 8.3 Hz, 1H), 4.32 – 4.07 (m, 4H), 3.78 (dq, J = 8.2, 4.0 Hz, 4H), 3.72 – 3.41 (m, 16H). **^{13}C NMR** (126 MHz, DMSO) δ 191.4, 153.6, 148.4, 129.6, 126.0, 112.3, 110.9, 70.4, 70.4, 70.3, 70.24, 70.19, 69.9, 69.8, 68.8, 68.7, 68.4. **HRMS (EI)** m/z calcd for $C_{19}H_{28}O_8$ $[M+H]^+$: 384.1777. Found: 384.1777. **LC-LRMS** Found: 385.8 ($M+H^+$), 407.8 ($M+Na^+$), 402.8 ($M+NH_4^+$), 423.8 ($M+K^+$).

III. Solution NMR

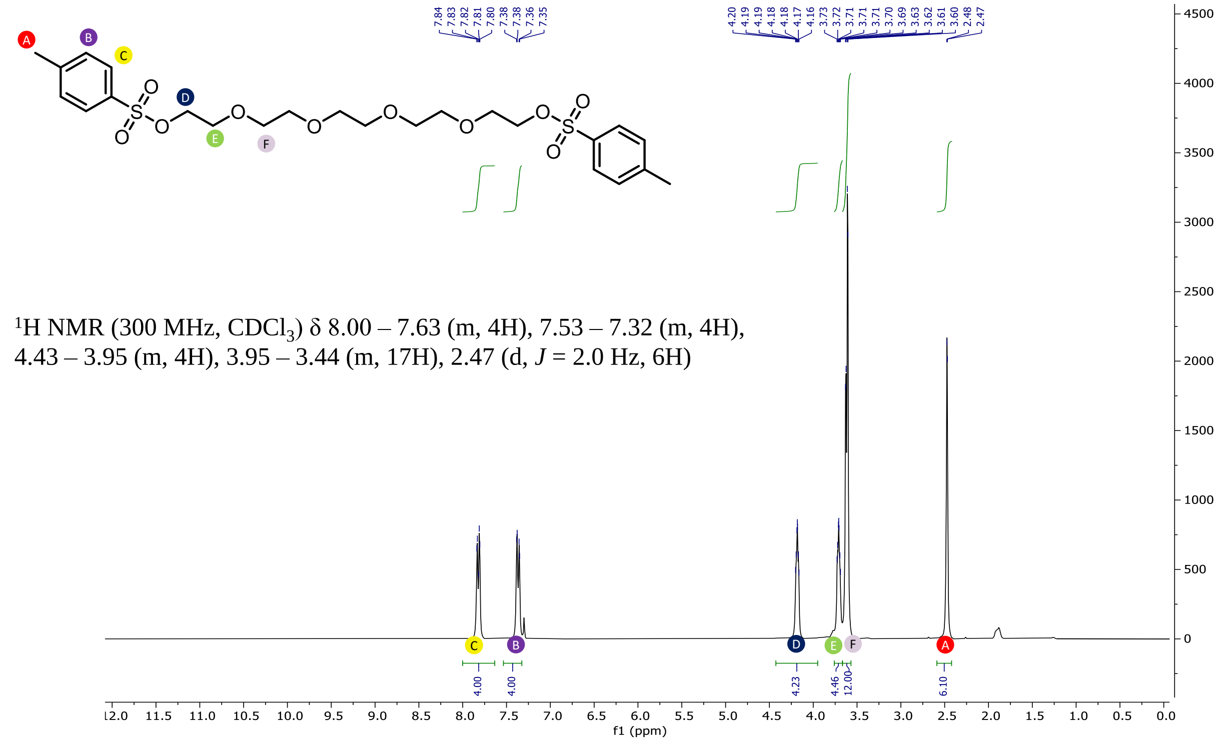

Figure S2. ^{1}H NMR ($CDCl_3$, 298 K) of 3,6,9,12-tetraoxatetradecane-1,14-diyl bis(4-methylbenzenesulfonate) **(2)**

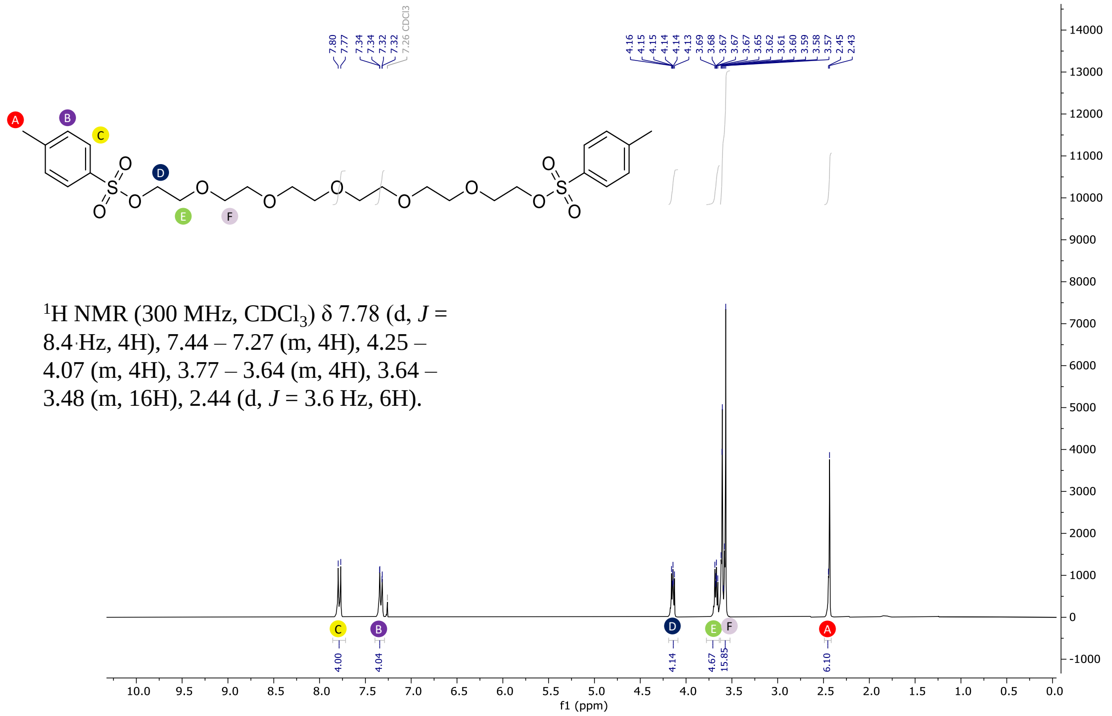

Figure S3. ^{1}H NMR ($CDCl_3$, 298 K) of 3,6,9,12,15-pentaoxaheptadecane-1,17-diyl bis(4-methylbenzenesulfonate) **(3)**

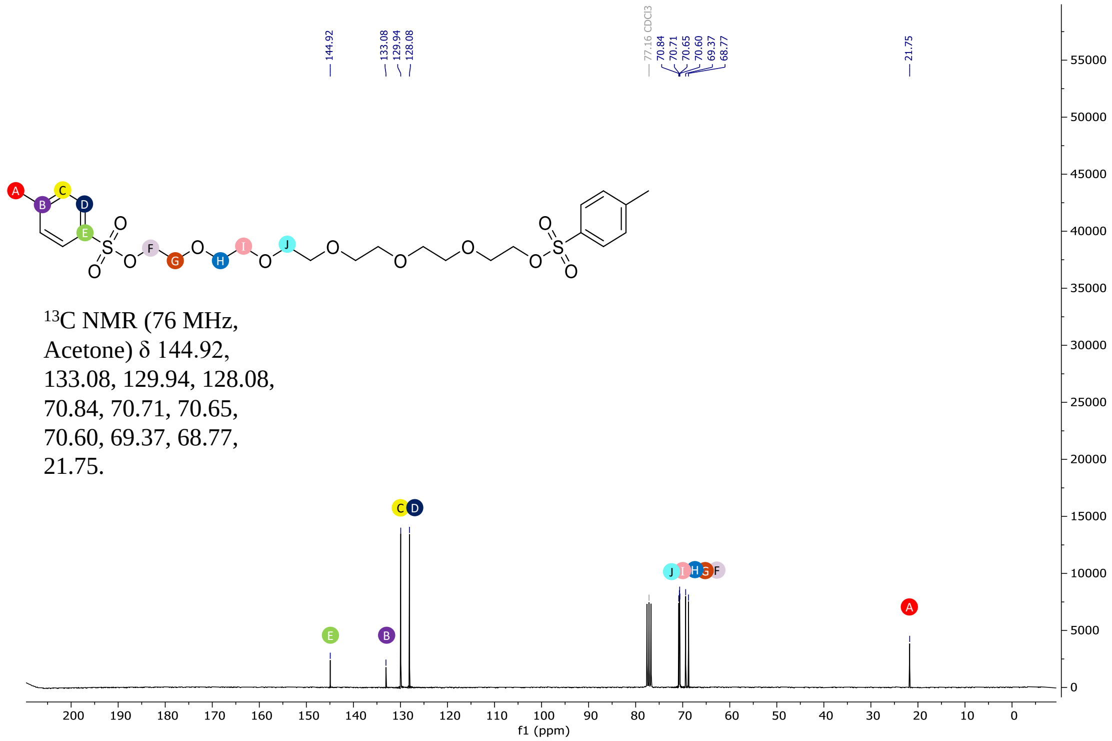

Figure S4. ^{13}C NMR (Acetone-D_6, 298 K) of 3,6,9,12,15,18-hexaoxaicosane-1,20-diyl bis(4-methylbenzenesulfonate) **(3).**

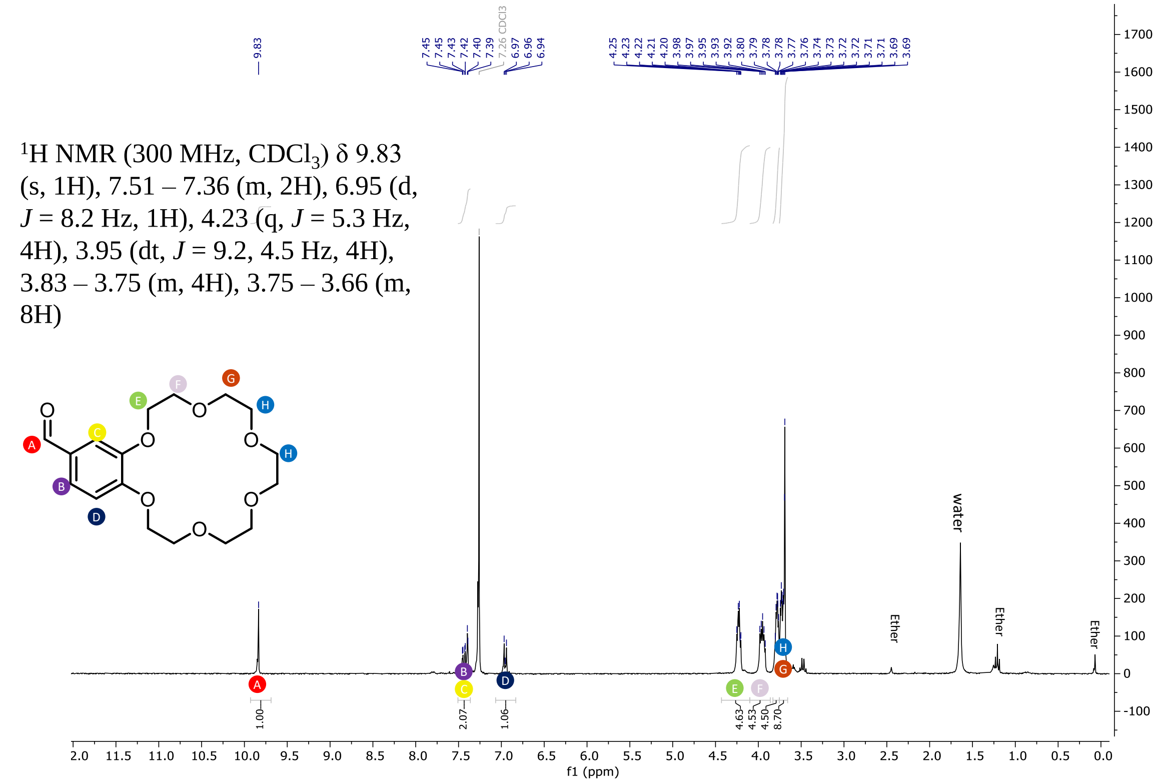

Figure S5. ^{1}H NMR ($CDCl_3$, 298 K) of 4'-Formylbenzo[18C6] **(C6).**

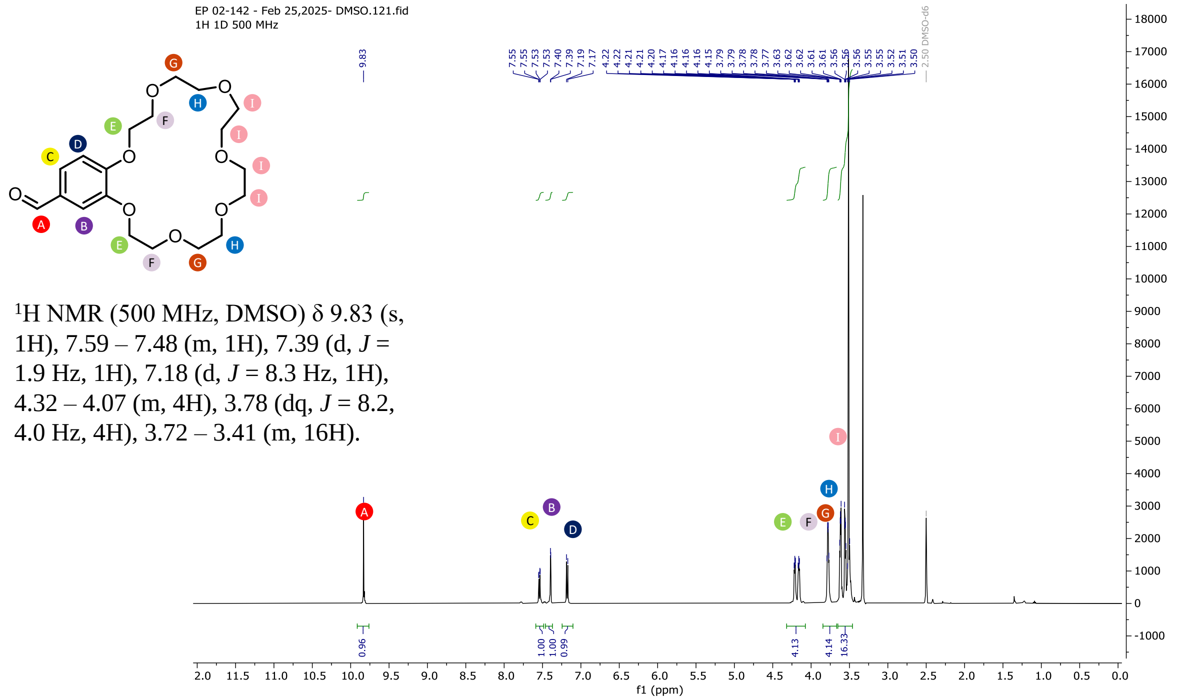

Figure S6. ¹H NMR (DMSO-D$_6$, 298 K) of 4’-Formylbenzo[21C7] **(C7).**

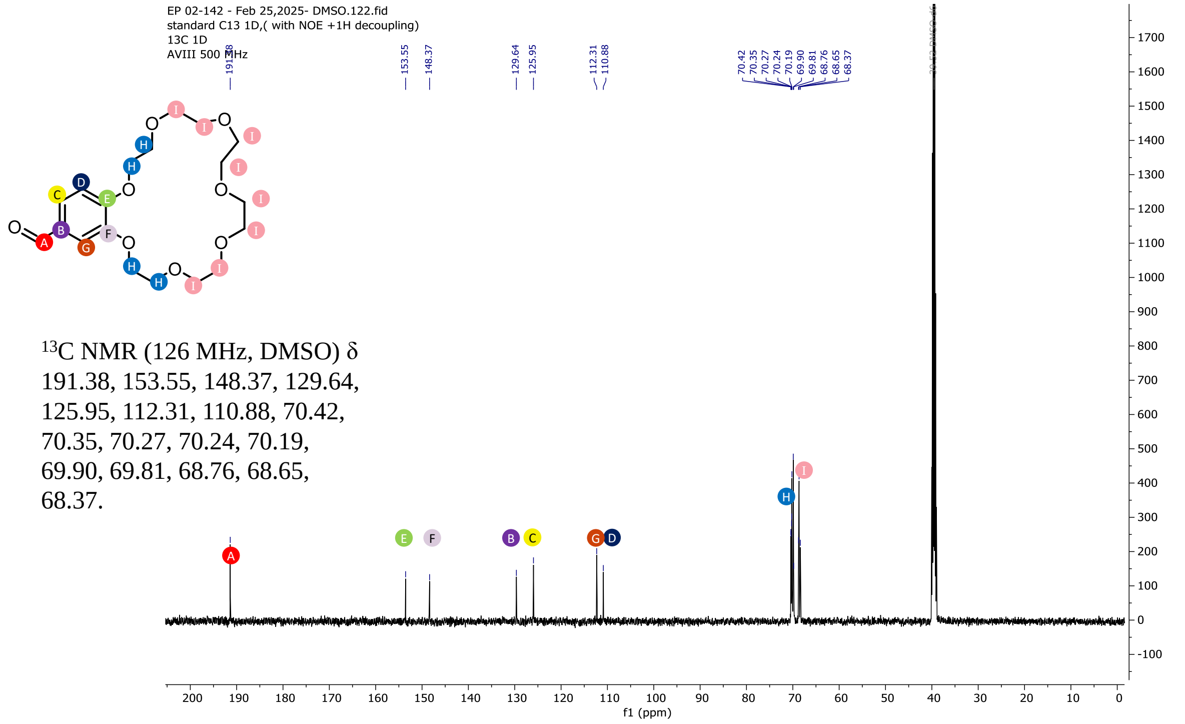

Figure S7. ¹³C NMR (DMSO-D$_6$, 298 K) of 4’-Formylbenzo[21C7] **(C7).**

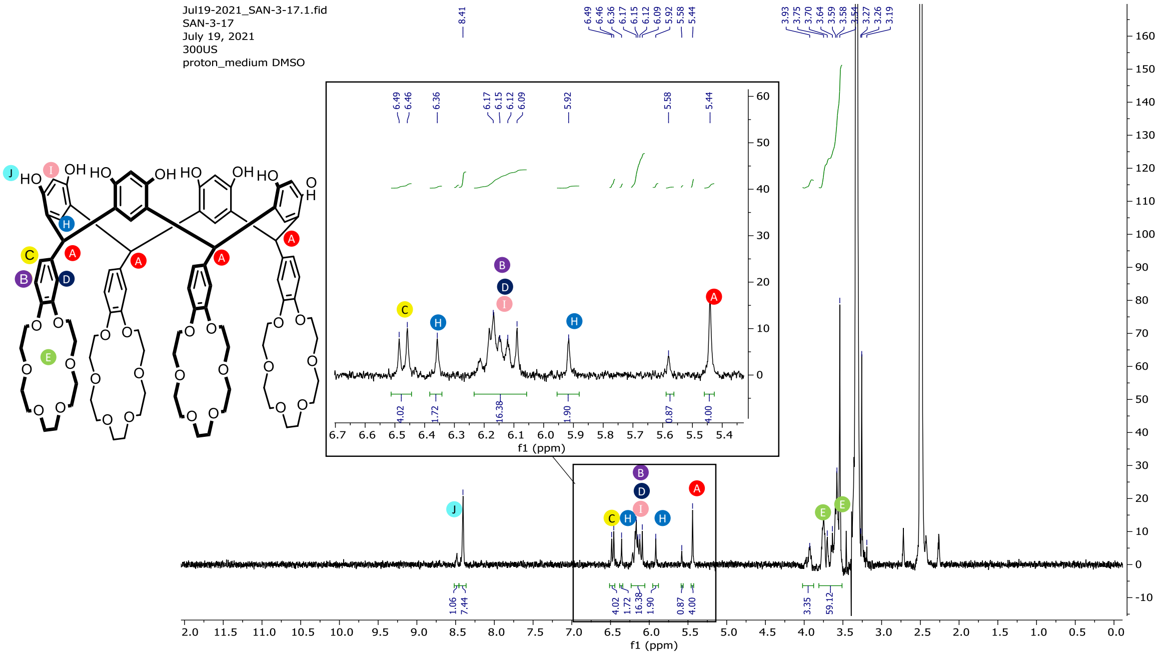

Figure S8. ^{1}H NMR (DMSO-D$_6$, 298 K) of **TRC6.**

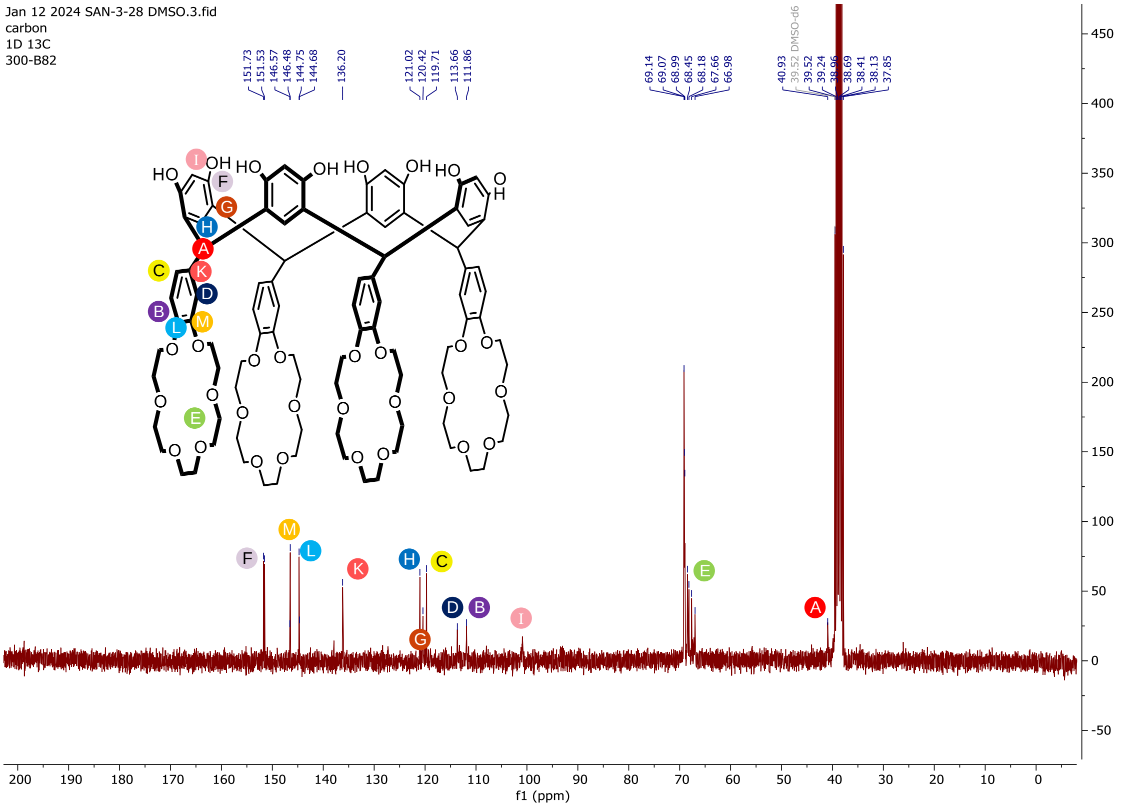

Figure S9. ^{13}C NMR (DMSO-D$_6$, 298 K) of **TRC6.**

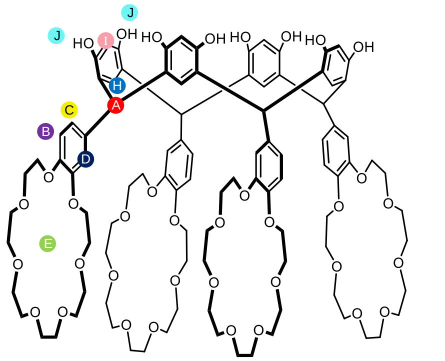

^{1}H NMR (500 MHz, DMSO) δ 8.40 (d, J = 11.4 Hz, 8H), 6.47 (d, J = 8.2 Hz, 4H), 6.35 (s, 2H), 6.26 – 6.02 (m, 12H), 5.87 (s, 2H), 5.45 (s, 4H), 3.94 (d, J = 3.8 Hz, 8H), 3.83 – 3.44 (m, 89H).

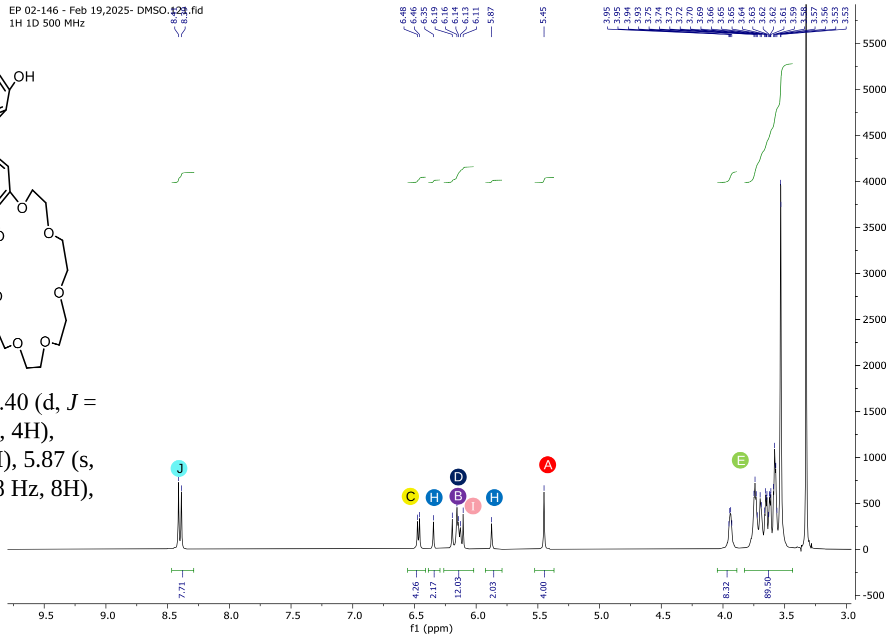

Figure S10. ^{1}H NMR (DMSO-D$_6$, 298 K) of **TRC7.**

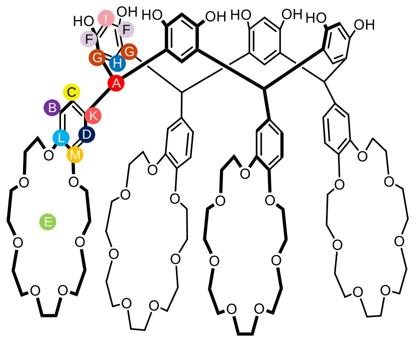

^{13}C NMR (126 MHz, DMSO) δ 152.50, 152.34, 147.21, 145.49, 137.11, 121.68, 121.20, 120.61, 114.56, 112.71, 101.70, 70.34, 70.32, 70.25, 70.19, 69.91, 69.85, 69.39, 69.06, 68.50, 67.88, 41.65.

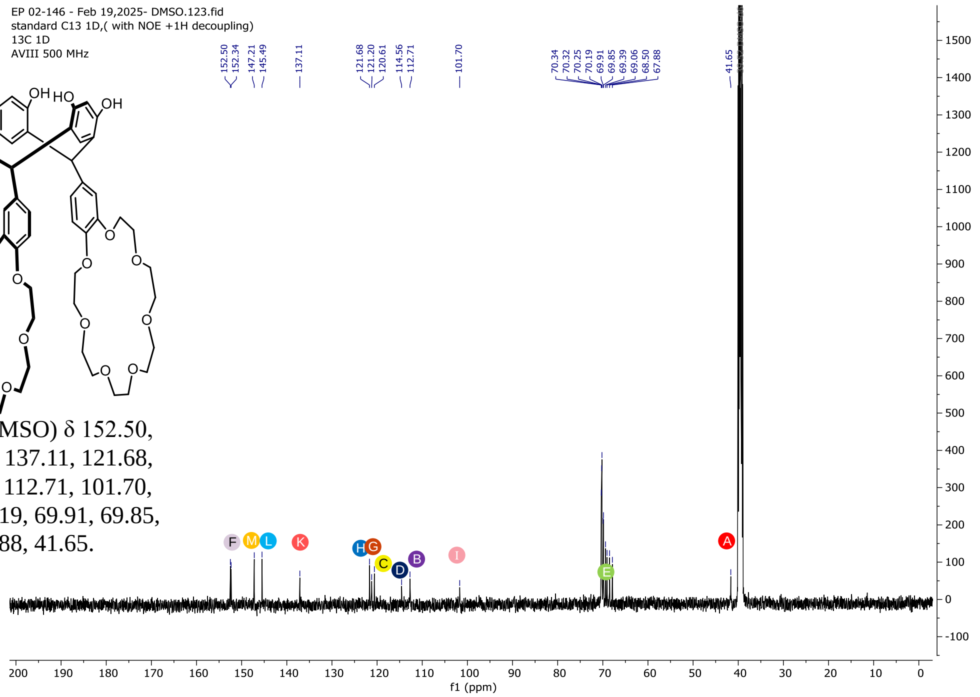

Figure S11. ^{13}C NMR (DMSO-D$_6$, 298 K) of **TRC7.**

IV. X-Ray crystallography Details

Refinement details:

TRC6 - Squeeze within Platon[5] was used to account for the unknown solvates present, which identified solvent accessible voids totaling 449.4 Å^3 with 138.2 electrons per unit cell being recovered. An estimate of the omitted solvates is: $2(C_3H_7NO)\cdot 6(H_2O)$.

TRC7 - Squeeze within Platon[5] was used to account for the unknown solvates present, which identified solvent accessible voids totaling 1560.5 Å^3 and 428.7 electrons per unit cell being recovered. An estimate of the omitted solvates is: $8(C_3H_7NO)\cdot 11(H_2O)$.

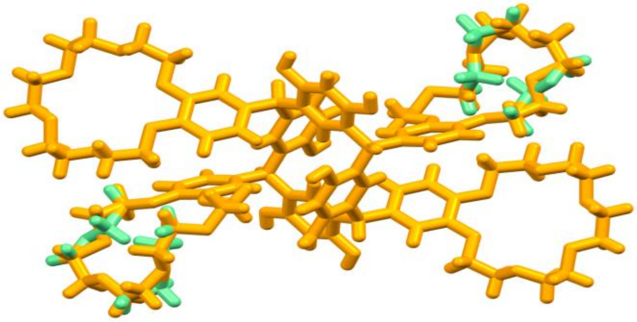

Figure S12. The disorder (50:50) of the second crown ethers in **TRC6**. All solvent molecules omitted for clarity.

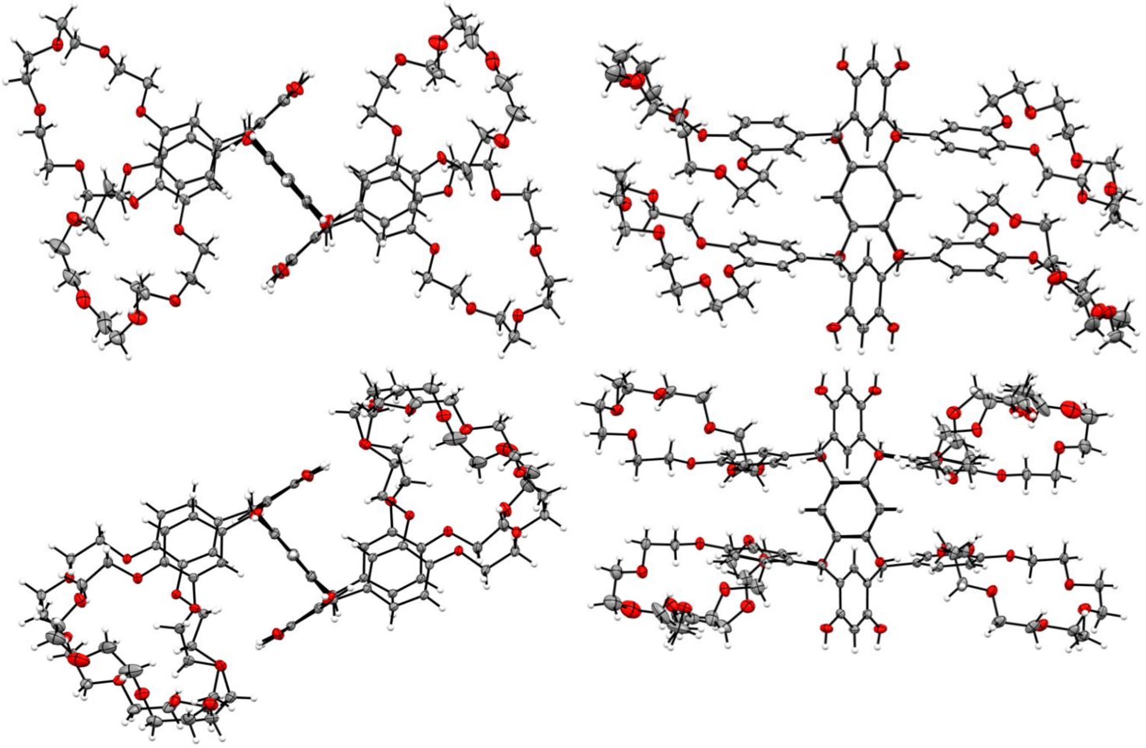

Figure S13. The ORTEP plots of **TRC6** (upper row) and **TRC7** (lower row)

Table S1 | Summary of crystal data and structure refinements.

**Complex**	**TRC6**	**TRC7**
Formula[§]	$C_{92}H_{112}O_{32}·4(C_3H_7NO)·4(H_2O)$	$C_{100}H_{128}O_{36}$
Formula Weight ($gmol^{-1}$)[§]	2094.26	1906.02
Color & Habit	Colorless needle	Colorless needle
Crystal Dimensions (mm)	0.03 × 0.03 × 0.25	0.04 × 0.06 × 0.25
Crystal System	Triclinic	Monoclinic
Space Group	*P*-1 (No. 2)	$P2_1/c$
a (Å)	13.3415(12)	11.42950(10)
b (Å)	16.1606(13)	24.4685(3)
c (Å)	16.3275(12)	21.4987(3)
α (°)	66.903(7)	90
β (°)	70.425(7)	98.4560(10)
γ (°)	74.146(8)	90
V (Å)	3009.9(5)	5947.02(12)
Z	1	2
ρ_{calc} (g/cm^3)[§]	1.155	1.064
F(000)[§]	1120	2032
μ (mm^{-1})	0.74	0.67
Temperature (K)	123.0(1)	100.0(1)
θ_{max} (°)	62.4	66.7
Total Reflections	14548	19053
Independent Reflections	9237	10433
Reflections ($I_o > 2\sigma[I_o]$)	6884	8829
R_{int}	0.0271	0.0234
Parameters	717	656
Restraints	2	3
GooF (F^2)	1.01	1.04
R_1 ($I_o > 2\sigma[I_o]$)	0.0571	0.0525
R_1 (all reflections)	0.0788	0.0605
wR_2 ($I_o > 2\sigma[I_o]$)	0.1466	0.1440
wR_2 (all reflections)	0.1655	0.1493
Largest Peak ($eÅ^{-3}$)	0.63	0.86
Largest Hole ($eÅ^{-3}$)	-0.43	-0.44
CCDC Number	2421650	2421651

§ These parameters have not been corrected for the unknown solvates accounted for using Squeeze within Platon.

V. Hirshfeld Surface Analysis

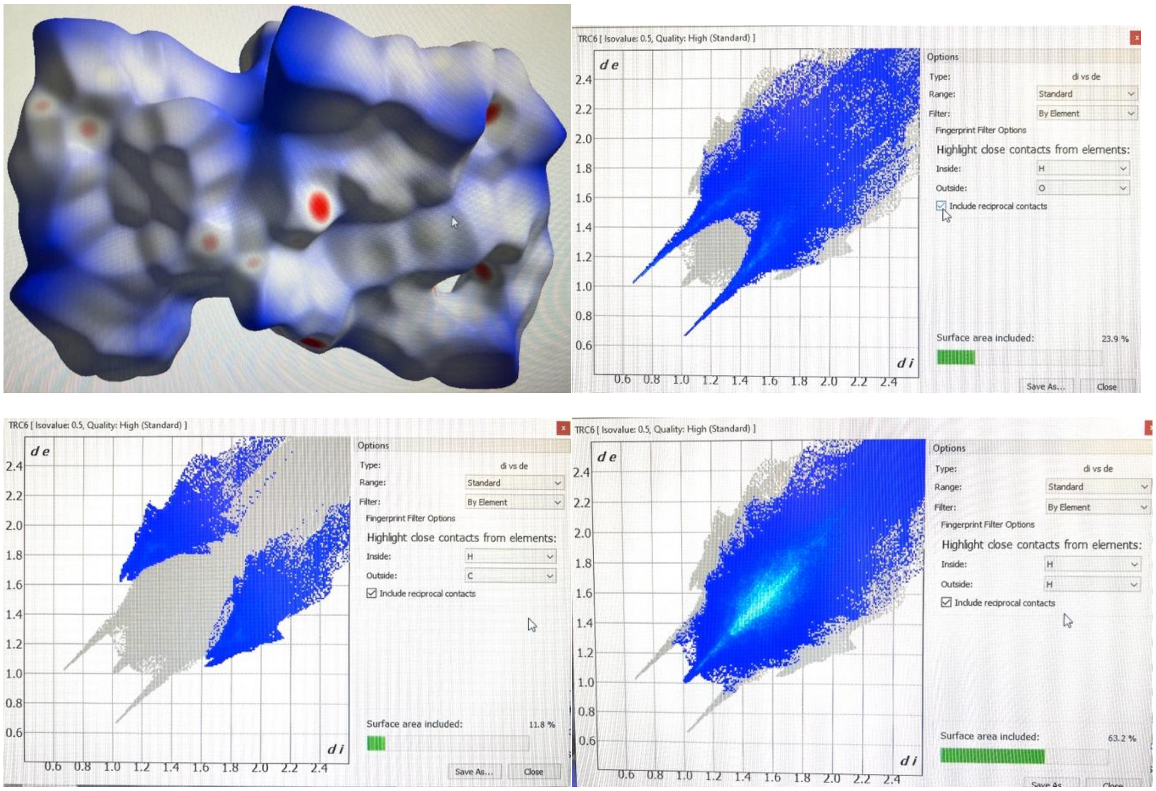

Figure S14. The Hirshfeld surface (upper left), the H…O interactions (upper right), the H…C interactions (lower left), and the H…H interaction (lower right) for **TRC6**.

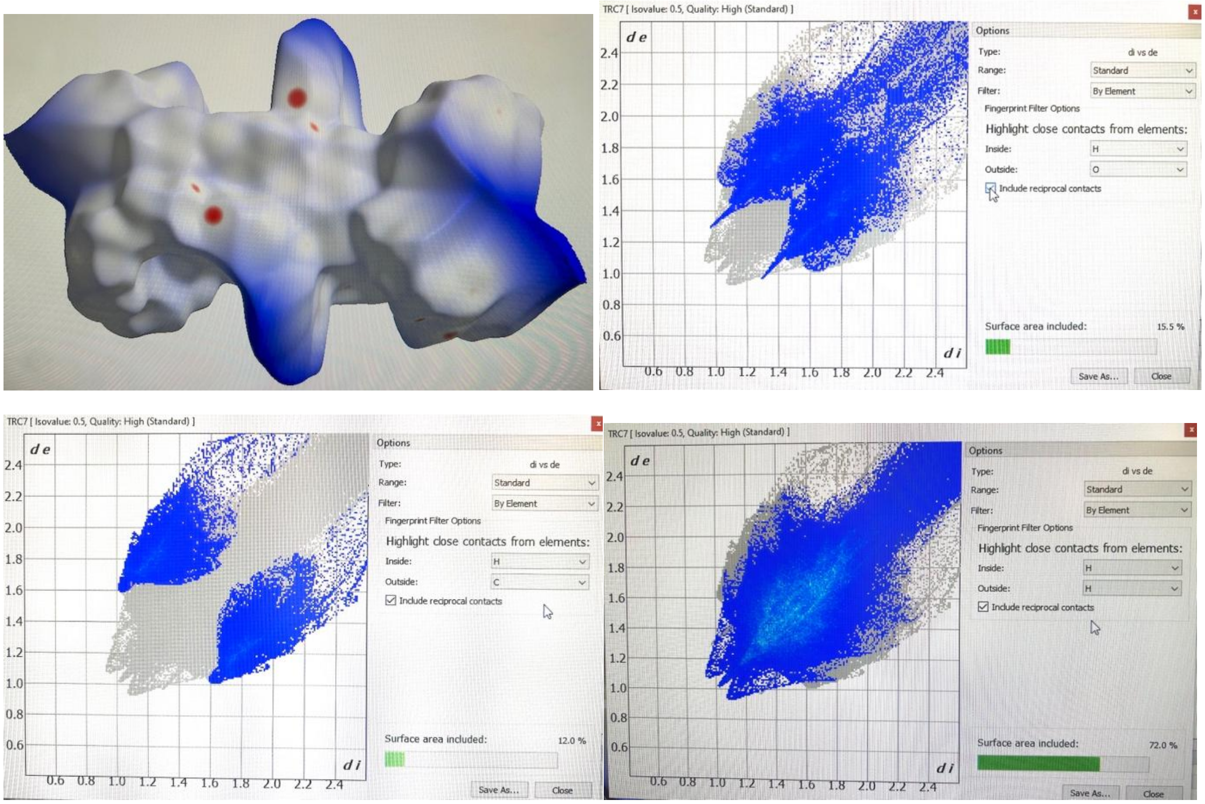

Figure S15. The Hirshfeld surface (upper left), the H…O interactions (upper right), the H…C interactions (lower left), and the H…H interactions (lower right) for **TRC7**.

VI. Complexation Studies

a) NMR Spectroscopy:

NMR spectra were recorded on a Bruker Avance DRX 400 spectrometer. All signals are given as δ values in ppm using residual solvent signals as the internal standard. A 1 mM concentration of each resorcinarene-crown ether and metallic salts was prepared in deuterated DMSO. Starting with 500 uL of pure resorcinarene-crown ether solution, NMR titration measurements were performed by replacing resorcinarene-crown ether solution with 50 uL incremental additions of metallic salt solution for different equivalents to make a 500 uL solution for each measurement. Job plots were made to derive stoichiometric parameters associated with the binding of **TRC6** and **TRC7** with K and Rb cations, respectively. Similar experiments were performed in acetonitrile with KBF4 and RbBF4. The chloride salts were not soluble in acetonitrile. Up to 5 equivalents of the salts from a 10 mM stock solution were added to a 1 mM sample solution of the resorcinarene crowns **TRC6** and **TRC7**.

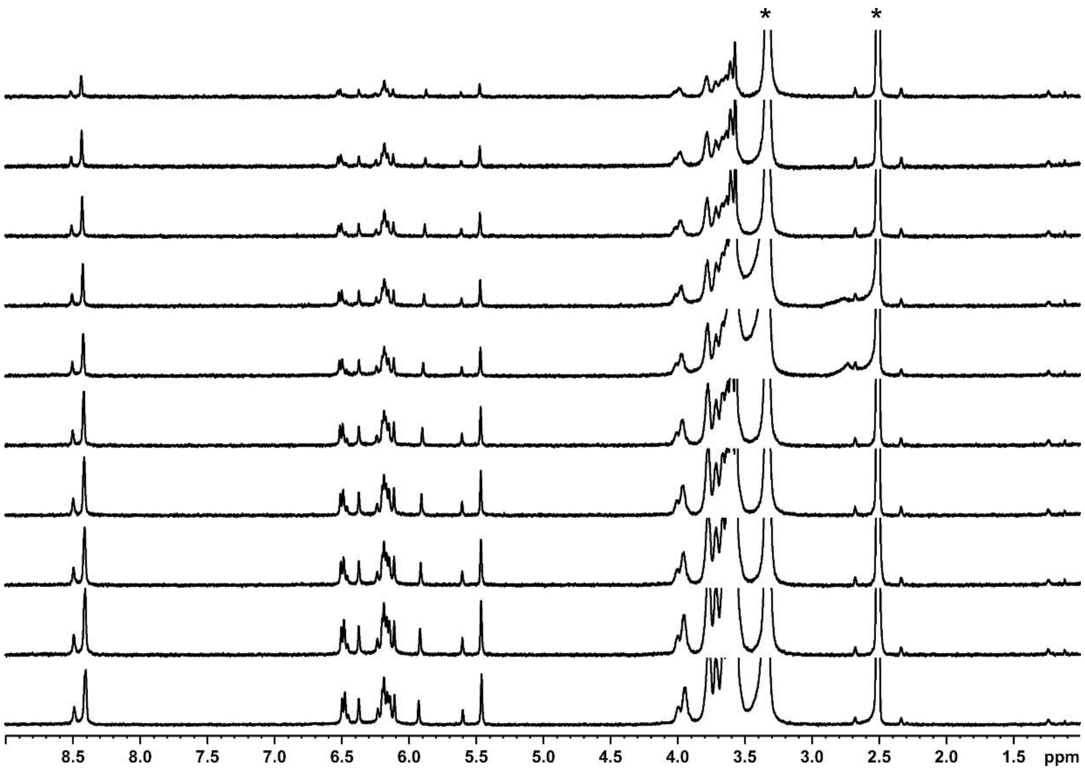

Figure S16: ^{1}H NMR stack spectra (DMSO-D_6, 298 K) showing the full spectra of pure **TRC6** and up to four equivalents of KCl. Star represents the residual solvent.

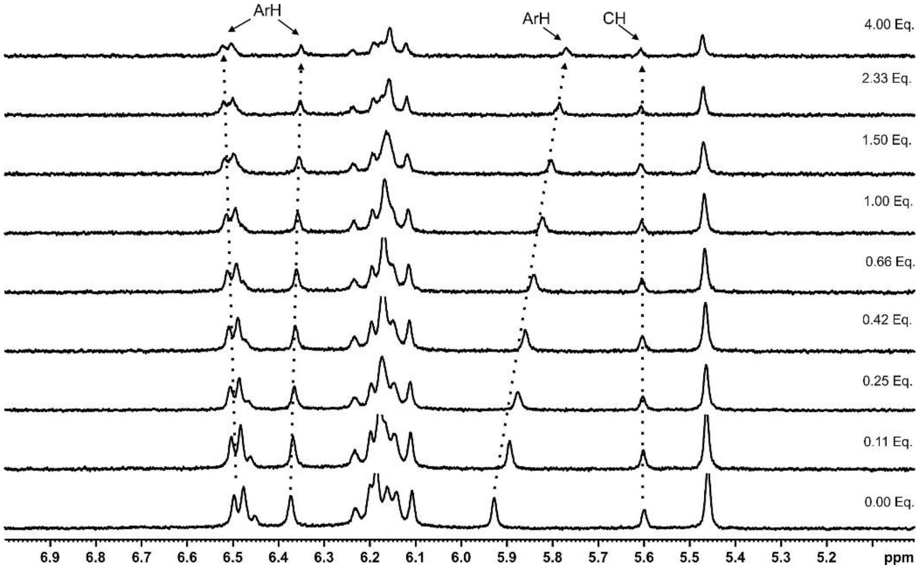

Figure S17: ^{1}H NMR stack spectra (DMSO-D_6, 298 K) showing pure **TRC6** and up to four equivalents of RbCl. The dash lines give an indication of the signal changes in ppm.

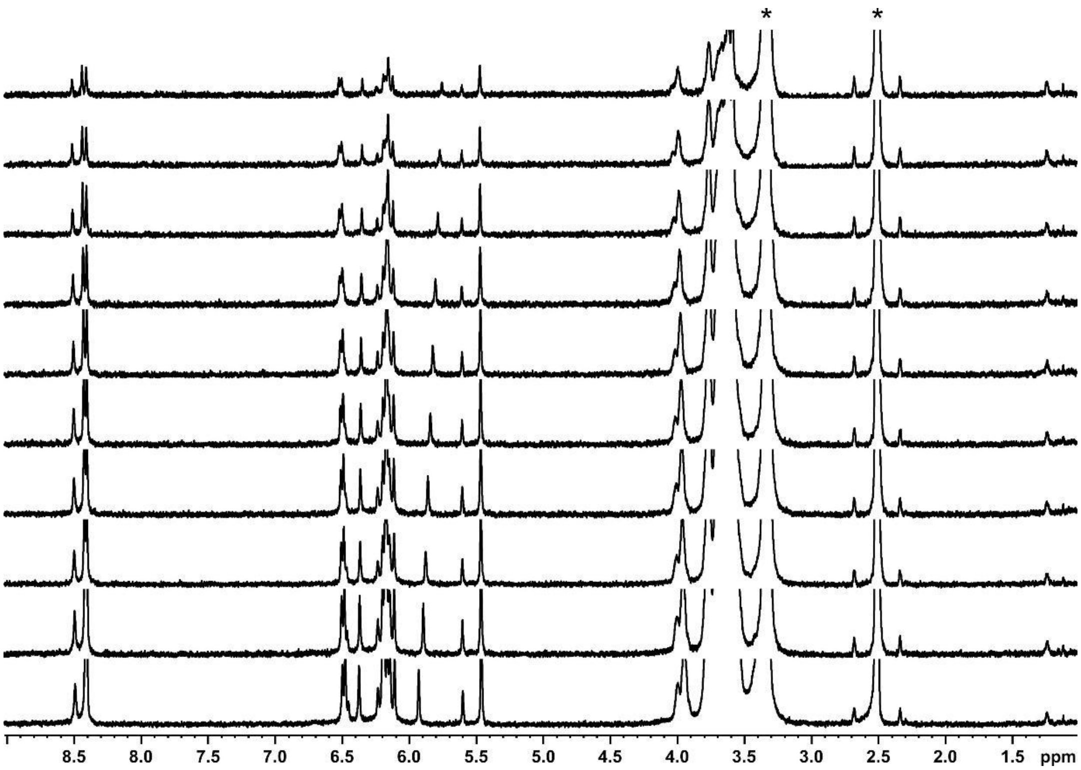

Figure S18: ^{1}H NMR stack spectra (DMSO-D$_6$, 298 K) showing the full spectra of pure **TRC6** and up to four equivalents of RbCl. Star represents the residual solvent.

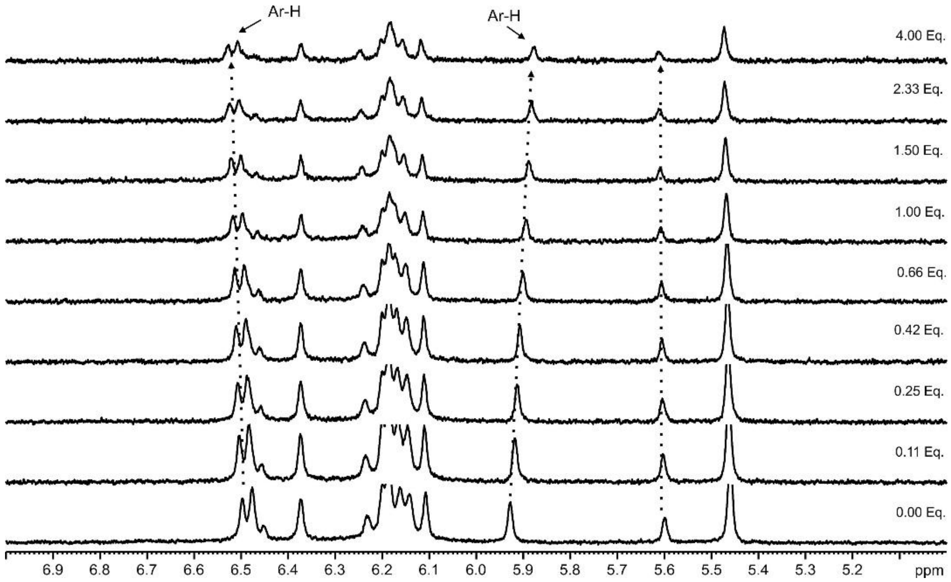

Figure S19: ^{1}H NMR stack spectra (DMSO-D$_6$, 298 K) showing pure **TRC7** and up to four equivalents of KCl. The dash lines give an indication of the signal changes in ppm.

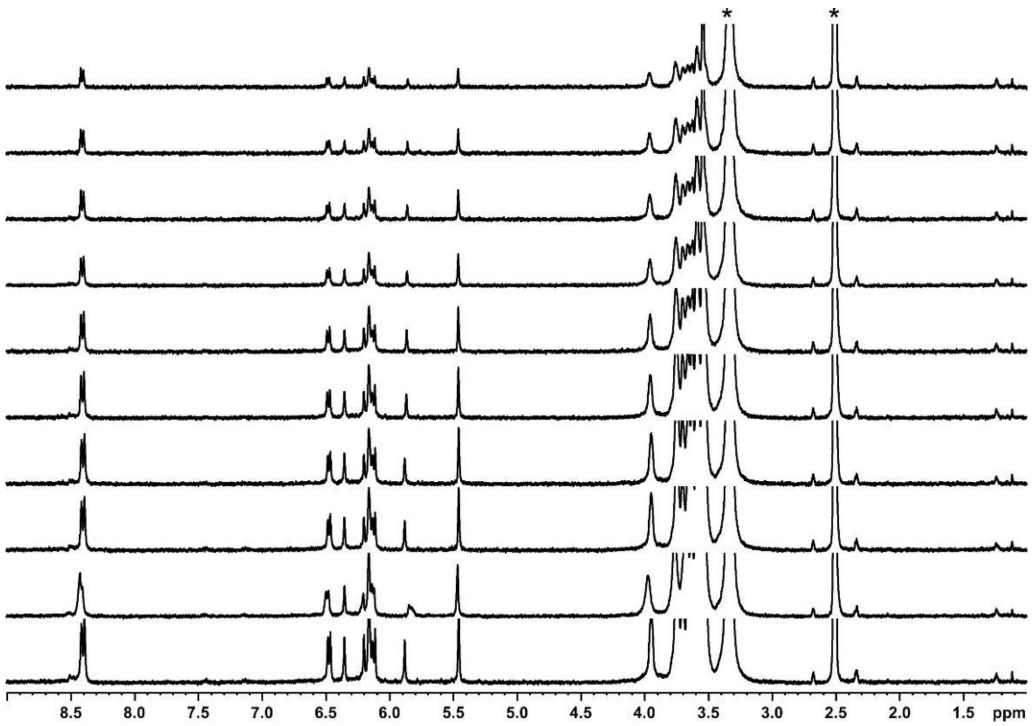

Figure S20: ^{1}H NMR stack spectra (DMSO-D_6, 298 K) showing the full spectra of pure **TRC7** and up to four equivalents of KCl. Star represents the residual solvent.

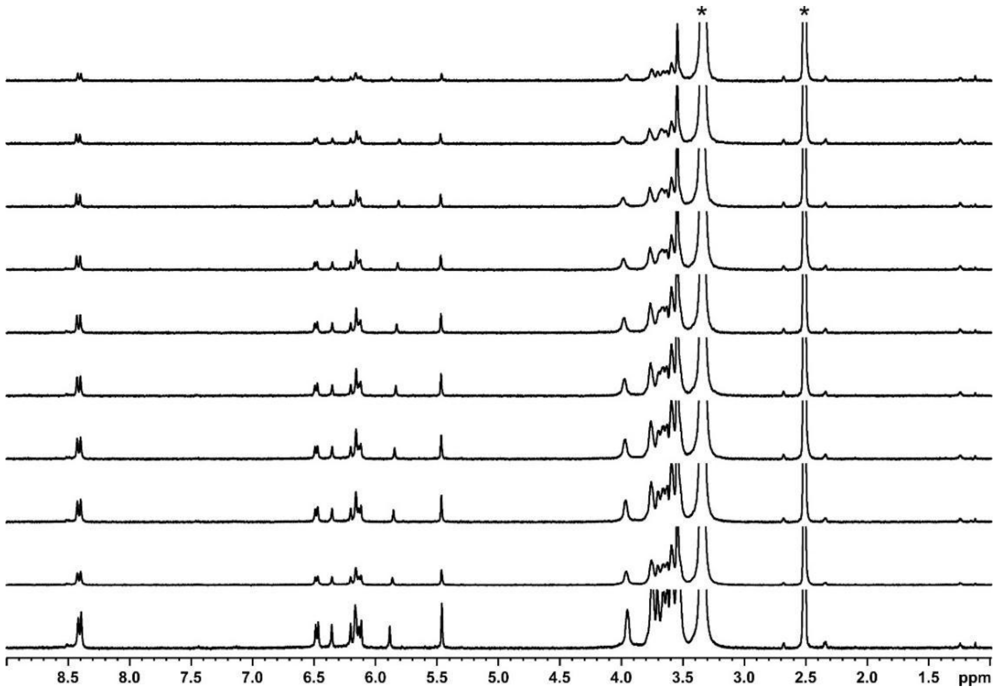

Figure S21: ^{1}H NMR stack spectra (DMSO-D_6, 298 K) showing the full spectra of pure **TRC7** and up to four equivalents of RbCl. Star represents the residual solvent.

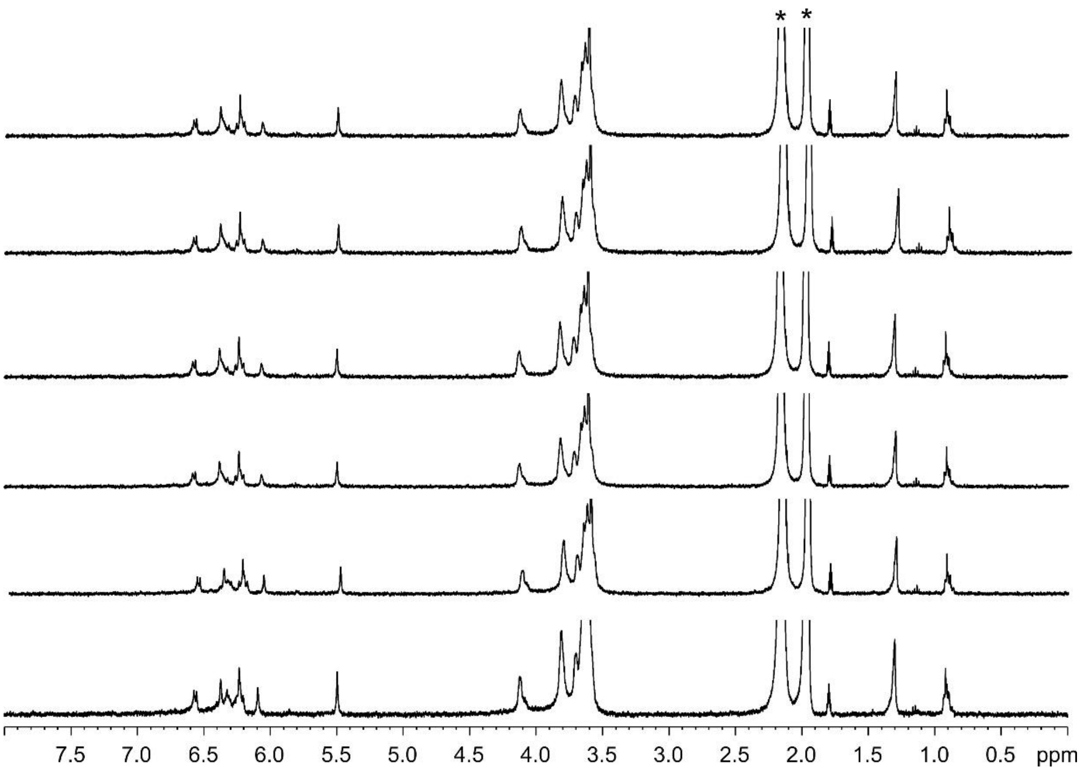

Figure S22: ^{1}H NMR stack spectra (CD_3CN, 298 K) showing the full spectra of pure **TRC6** and up to five equivalents of KBF_4. Stars represent the residual solvent.

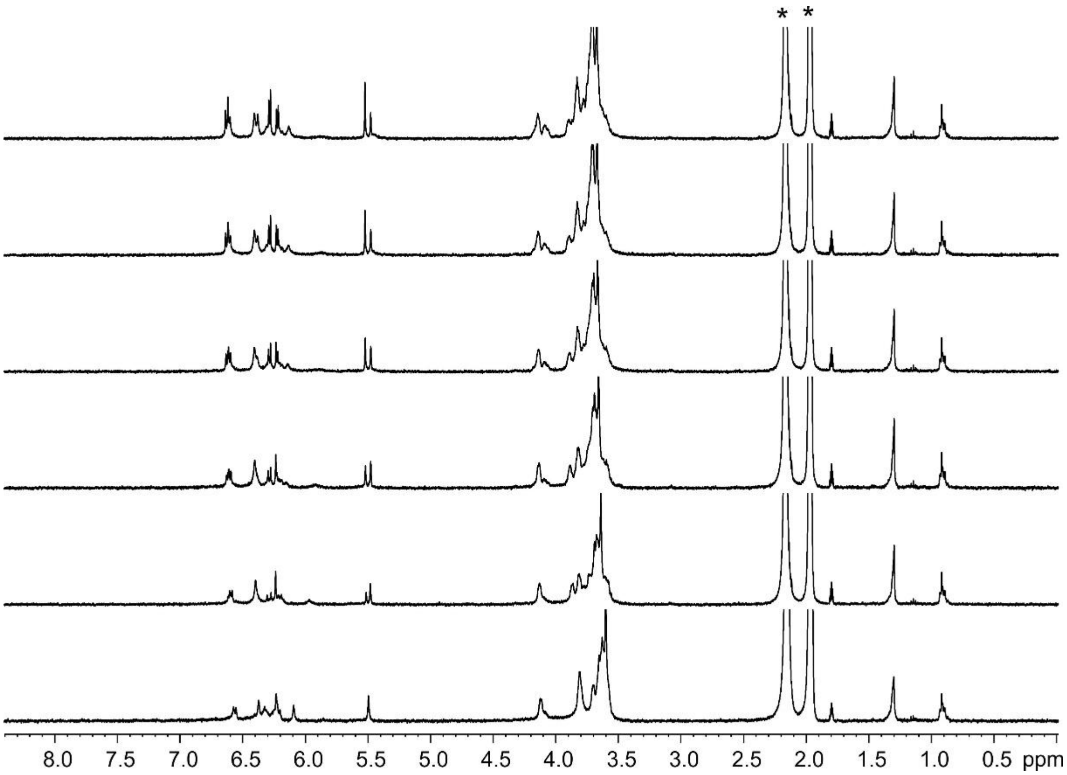

Figure S23: ^{1}H NMR stack spectra (CD_3CN, 298 K) showing the full spectra of pure **TRC6** and up to five equivalents of $RbBF_4$. Stars represent the residual solvent.

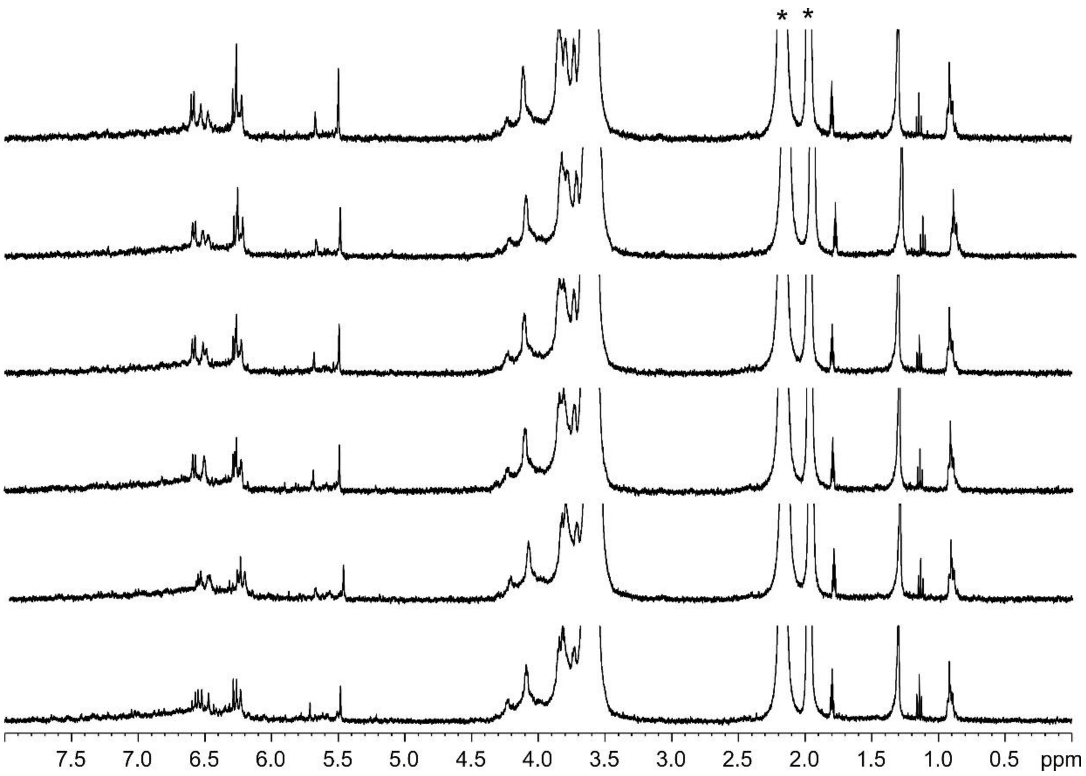

Figure S24: ^{1}H NMR stack spectra (CD_3CN, 298 K) showing the full spectra of pure **TRC7** and up to five equivalents of KBF_4. Stars represent the residual solvent.

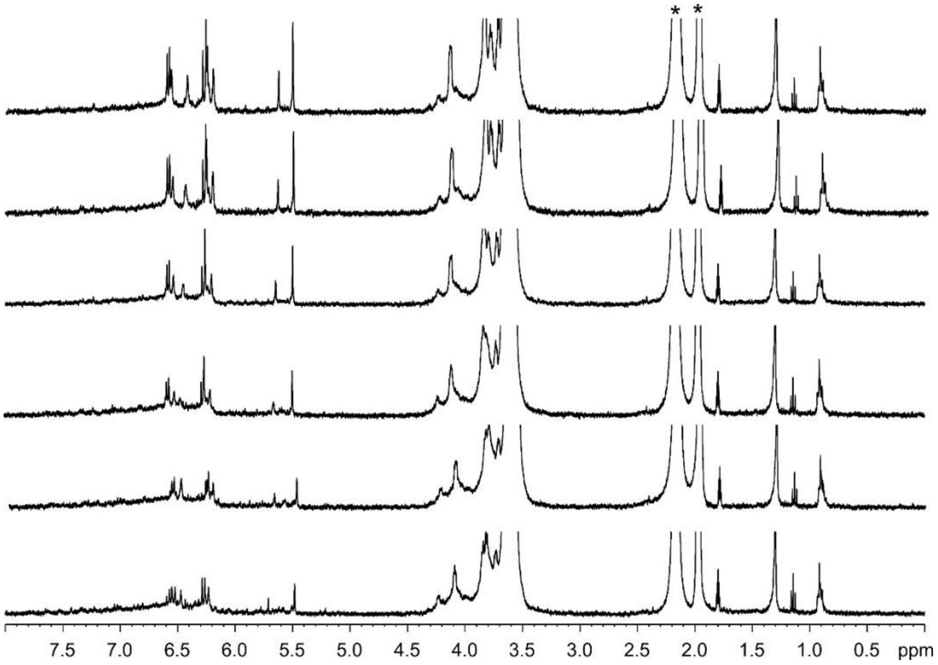

Figure S25: ^{1}H NMR stack spectra (CD_3CN, 298 K) showing the full spectra of pure **TRC7** and up to five equivalents of $RbBF_4$. Stars represent the residual solvent.

b) Job Plots

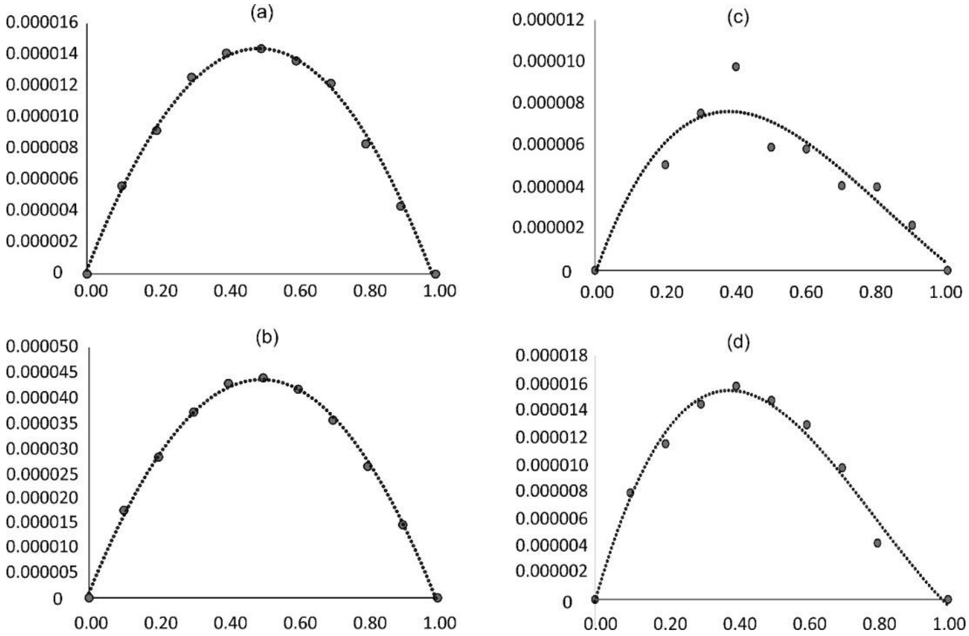

Figure S26: Job's plot (DMSO-D_6, 298 K) showing the suggested stoichiometry of (a) **TRC6** and KCl (1:1), (b) **TRC6** and RbCl (1:1), (c) **TRC7** and KCl (1:2), and (d) **TRC7** and RbCl (1:2).

c) Isothermal Titration Calorimetry (ITC):

A NaNoITC instrument by TA Instruments was used to determine the molar enthalpy (ΔH) of complexation. Subsequent fitting of the data into a sequential binding model provides the association constants (Ka), change in enthalpy (ΔH), and entropy (ΔS). The ITC experiment was carried out by filling the sample cell with one sample (substrate, 1 mM), filling the syringe with the second sample (titrant, 10 mM), and titrating via computer-automated injector at 298 K. Blank titrations into plain solvent were also performed and subtracted from the corresponding titration to remove any effect from the heats of dilution from the titrant.

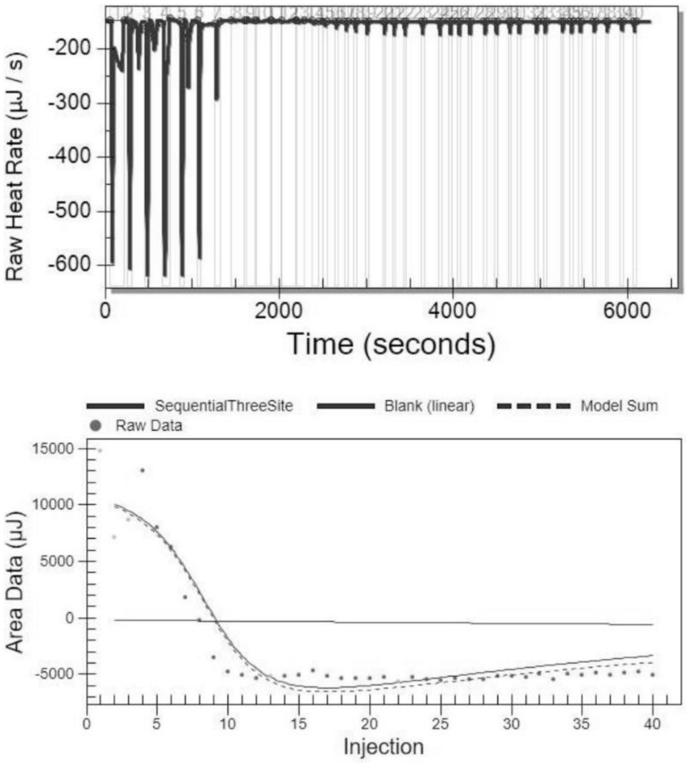

Figure S27: Isothermal calorimetric titration isotherms of titrating **KBF_4** (10 mM) into **TRC6** (1 mM) in acetonitrile at 298K

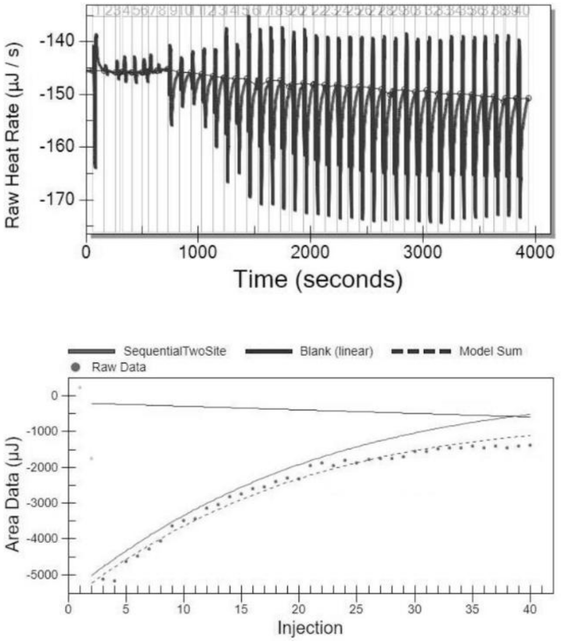

Figure S28: Isothermal calorimetric titration isotherms of titrating **$RbBF_4$** (10 mM) into **TRC6** (1 mM) in acetonitrile at 298K

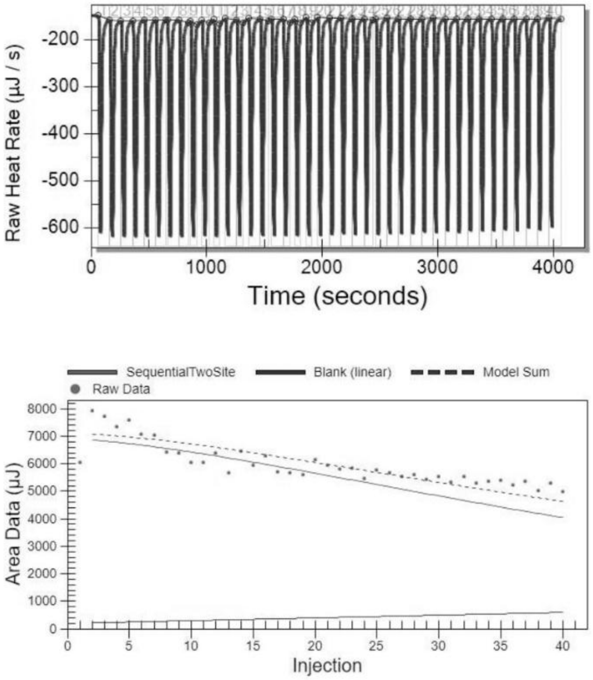

Figure S29: Isothermal calorimetric titration isotherms of titrating **KBF_4** (10 mM) into **TRC7** (1 mM) in acetonitrile at 298K

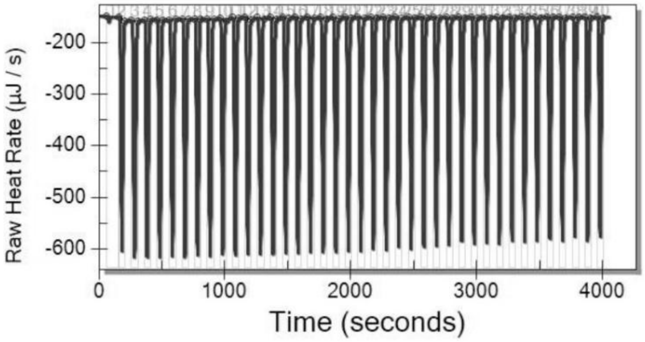

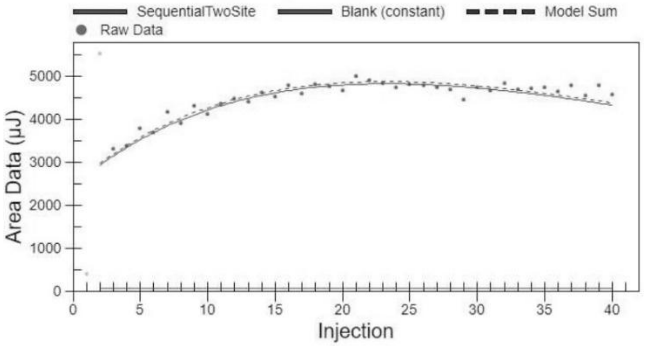

Figure S30: Isothermal calorimetric titration isotherms of titrating **$RbBF_4$** (10 mM) into **TRC7** (1 mM) in acetonitrile at 298K

VI. References

1. Timmerman, P., Verboom, W. & Reinhoudt, D. N. Resorcinarenes. *Tetrahedron* **52**, 2663–2704 (1996).
2. Chen, Y. & Baker, G. L. Synthesis and properties of ABA amphiphiles. *Journal of Organic Chemistry* **64**, 6870–6873 (1999).
3. Fujino, T. *et al.* Formation of [2]- and [3]Rotaxanes through Bridging under Kinetic and Thermodynamic Control. *Organic Letters* **20**, 369–372 (2018).
4. D'Souza, F. *et al.* Design, syntheses, and studies of supramolecular porphyrin-fullerene conjugates, using bis-18-crown-6 appended porphyrins and pyridine or alkyl ammonium functionalized fullerenes. *Journal of Physical Chemistry* **110**, 5905–5913 (2006).
5. Spek, A. L. PLATON SQUEEZE: a tool for the calculation of the disordered solvent contribution to the calculated structure factors. *Acta Crystallographica Section C: Structural Chemistry* **71**, 9–18 (2015).
